# 4-vinyl-substituted pyrimidine nucleosides exhibit the efficient and selective formation of interstrand cross-links with RNA and duplex DNA

**DOI:** 10.1093/nar/gkt197

**Published:** 2013-06-18

**Authors:** Atsushi Nishimoto, Daichi Jitsuzaki, Kazumitsu Onizuka, Yosuke Taniguchi, Fumi Nagatsugi, Shigeki Sasaki

**Affiliations:** ^1^Graduate School of Pharmaceutical Sciences, Kyushu University, Fukuoka 812-8582, Japan, ^2^CREST, Japan Science and Technology Agency, Kawaguchi, Saitama 332-0012, Japan and ^3^Institute of Multidisciplinary Research for Advanced Materials, Tohoku University, Sendai, Miyagi 980-8577, Japan

## Abstract

The formation of interstrand cross-links in nucleic acids can have a strong impact on biological function of nucleic acids; therefore, many cross-linking agents have been developed for biological applications. Despite numerous studies, there remains a need for cross-linking agents that exhibit both efficiency and selectivity. In this study, a 4-vinyl-substituted analog of thymidine (T-vinyl derivative) was designed as a new cross-linking agent, in which the vinyl group is oriented towards the Watson–Crick face to react with the amino group of an adenine base. The interstrand cross-link formed rapidly and selectively with a uridine on the RNA substrate at the site opposite to the T-vinyl derivative. A detailed analysis of cross-link formation while varying the flanking bases of the RNA substrates indicated that interstrand cross-link formation is preferential for the adenine base on the 5′-side of the opposing uridine. In the absence of a 5′-adenine, a uridine at the opposite position underwent cross-linking. The oligodeoxynucleotides probe incorporating the T-vinyl derivative efficiently formed interstrand cross-links in parallel-type triplex DNA with high selectivity for dA in the homopurine strand. The efficiency and selectivity of the T-vinyl derivative illustrate its potential use as a unique tool in biological and materials research.

## INTRODUCTION

Many chemical entities, of either exogenous or endogenous origins, cause the alkylation of or damage to DNA and RNA; thus, they have a strong impact on biological functions of nucleic acids (1–3). Chemotherapeutic agents, such as mitomycin C, exert their effects by the alkylation of DNA (4). On UV irradiation, psoralen forms DNA adducts with both duplex strands to form an interstrand cross-link. This compound is widely used for therapy (5) and mechanistic studies of processes, such as DNA repair (6). The enhanced inhibition of translation by antisense oligodeoxynucleotides (ODNs) has been demonstrated by the cross-link formation between psoralen and target RNA (7,8). Cross-link formation is also used to maintain 3D nucleic acid structures (9–11). A variety of functional groups have also been developed to enable interstrand cross-linking, including disulfide bonds (12), benzophenone derivatives (13), carbazoles (14), quinone methides (15,16), phenylselenyl derivatives of pyrimidines (17) and furan derivatives (18). To further advance these studies, an efficient cross-linking method is still desired.

**Figure 1.**
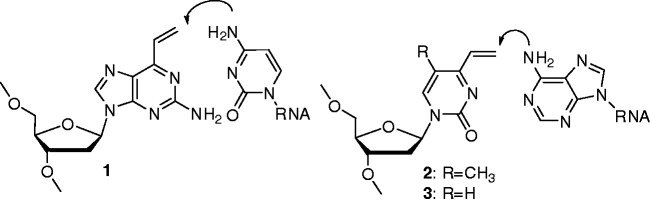
The 2-amino-6-vinylpurine derivative (**1**) for cross-linking with cytosine and newly designed 4-vinyl substituted pyrimidine derivatives, T-vinyl (**2**) and U-vinyl (**3**).

To address the need for an efficient cross-linking agent, we previously developed a 2-amino-6-vinylpurine derivative (**1**) based on a hybridization-assisted strategy. This compound exhibited efficient and selective cross-linking to cytosine bases (19–25). The close proximity of the vinyl group to the cytosine base in the hybridized complex contributed to the high reactivity of **1** (Figure 1). An ODN incorporating a sulfide-protected derivative of 2-amino-6-vinylpurine was shown to be useful for inhibiting and modulating intracellular gene expression (26). A characteristic feature of this 2-amino-6-vinylpurine is that the vinyl group is directed towards the Watson–Crick base pairing face to react with the 4-amino group of cytosine. Generally, the reactive group for cross-link formation is not located near the Watson–Crick face. The efficient cross-link formation by **1** suggests that partial base pairing and/or shape complementarity can benefit from a proximity effect. Further efforts have continued towards the biological applications and improvement of the 2-amino-6-vinylpurine unit (27–30). In this study, newly designed 4-vinylpyrimidine-2-one nucleoside analogs (T-vinyl **2** and U-vinyl **3**) have been shown to exhibit fast and selective cross-link formation with either adenine or uracil bases, depending on the target sequence. Herein, we describe in detail the synthesis of nucleoside analogs **2** and **3**, evaluation of their cross-linking reactivity, analysis of the cross-linked products derived from **2** and highlight its potential application for cross-linking in triplex DNA.

## MATERIALS AND METHODS

**Scheme 1.**
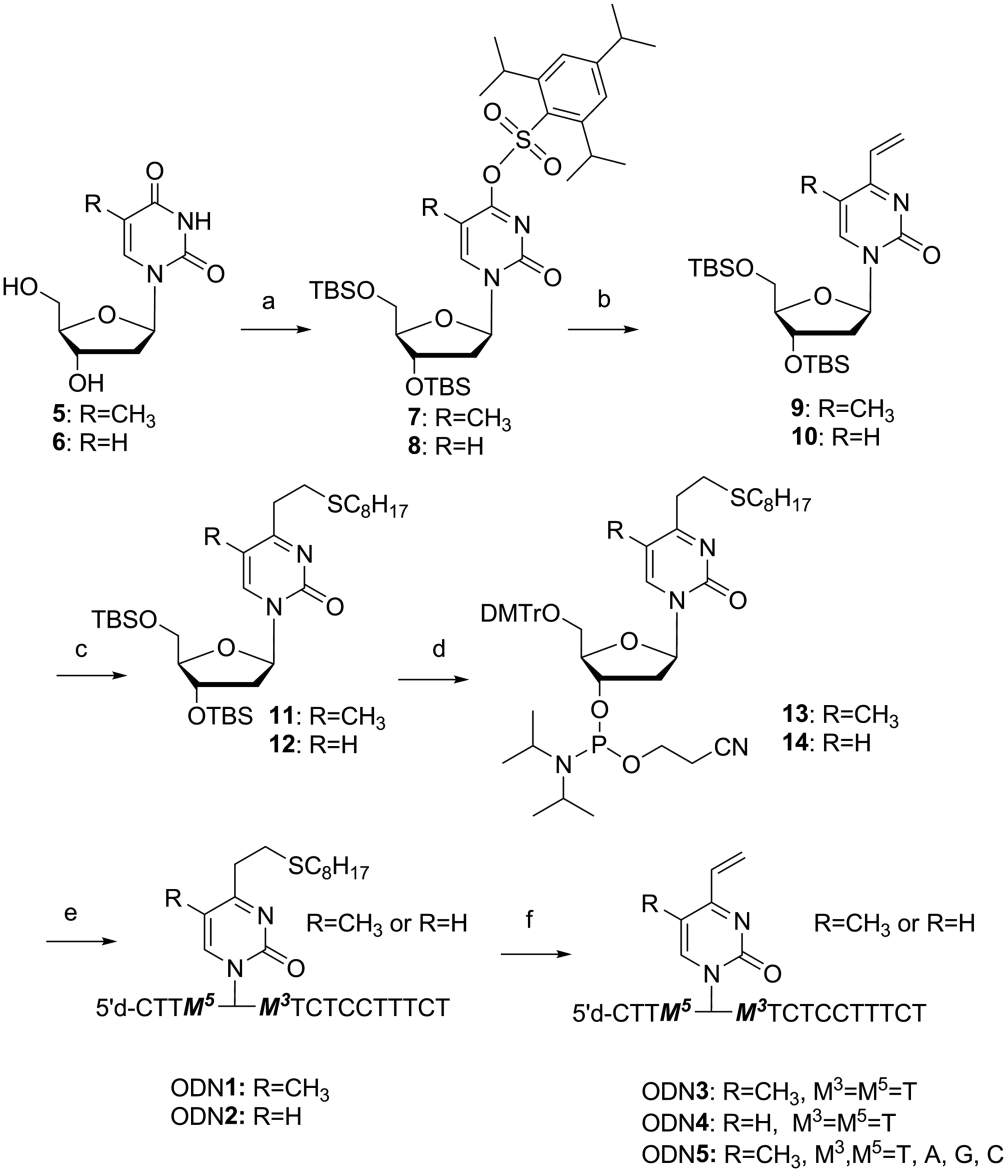
Synthesis of 4-vinyl-(*1H*)-pyrimidine-2-one derivatives (*2* and 3)^a^. (a) (i) TBDMSCl, imidazole, N,N-dimethylformamide, 94% and (ii) 2,4,6-triisopropylbenzensulfonyl chloride, Et_3_N, N,N-dimethyl-4-aminopyridine, CH_2_Cl_2_, 93%; (b) 2,4,6-trivinylcyclotriboroxane pyridine complex, Pd(PPh_3_)_4_, LiBr, K_2_CO_3_, H_2_O:1,4-dioxane = 1:3 solution; (c) C_8_H_17_SH, CH_3_CN, 94% (2 steps); (d) (i) tetrabutylammonium fluoride, tetrahydrofuran, 79%, (ii) DMTrCl, thioanisole, N,N-Diisopropylethylamine (DIPEA), CH_2_Cl_2_, 88% and (iii) 2-cyanoethyl *N,N*-diisopropylchlorophosphoramidite, DIPEA, CH_2_Cl_2_, 72%; (e) (i) DNA/RNA synthesizer, (ii) K_2_CO_3_ in dry methanol in the presence of octanethiol and (iii) 5% aqueous AcOH; and (f) (i) MMPP carbonate buffer, and (ii) 0.5 M NaOH.

The 4-vinyl-(*1H*)-5-methylpyrimidine-2-one derivative (**2**) was designed for cross-link formation with the 6-amino group of adenine, based on the expectation that the two bases would exhibit shape-complementarity resembling a T–A base pair. A similar derivative lacking the 5-methyl group (**3**) was also synthesized for comparison. The synthesis of the nucleoside units and their incorporation into ODNs are shown in Scheme 1. The syntheses described herein are an extension of earlier reports from this group concerning the introduction of a vinyl group via Suzuki–Miyaura coupling. The 2,4,6-triisopropylbenzensulfonyl derivative of tert-butyldimethylsilyl (TBDMS)-protected thymidine (**7**) or 2′-deoxyuridine (**8**) was treated with 2,4,6-trivinylcyclotriboroxane pyridine complex in the presence of Pd(PPh_3_)_4_, LiBr and K_2_CO_3_ in H_2_O-dioxane. As the resulting vinylated products (**9** and **10**) were not sufficiently stable for isolation, they were isolated after protection with octanethiol (**11** and **12**) (31). They were then converted to the corresponding phosphoramidite precursors (**13** and **14**) using conventional methods and were then used in a DNA/RNA automated synthesizer to incorporate **2** or **3** into ODN**1** and ODN**2** with flanking bases (*M^5^* and *M^3^*). Initially, vinyl derivative (**9**) was protected with methanethiol. However, the methylsulfide protecting group was cleaved during the reaction with DMTrCl and resulted in a complex mixture. This problem was overcome by protecting the vinyl group with octanethiol. Additionally, thioanisole was required as a scavenger for the DMTr cation during protection with DMTrCl. The ODNs thus synthesized were cleaved from the resin by treatment with a solution of K_2_CO_3_ in dry methanol in the presence of octanethiol. After HPLC purification, the DMTr group was deprotected in 5% aqueous AcOH solution, and the deprotected ODNs were again purified using HPLC to produce the octylsulfide-protected ODNs (**1** and **2**). The octylsulfide group was oxidized with magnesium metaperoxyphthalate (MMPP) in carbonate buffer, and the resulting sulfoxide derivatives were treated with 0.5 M NaOH solution to generate the vinyl group, thus providing the cross-linking ODNs (**3**,**4** and **5**) in good yields. The vinyl group of ODN was hydrated with a half-life of ∼4 h at 25°C and pH 7.0.

## RESULTS AND DISCUSSION

### Evaluation of the cross-linking reaction

**Figure 2.**
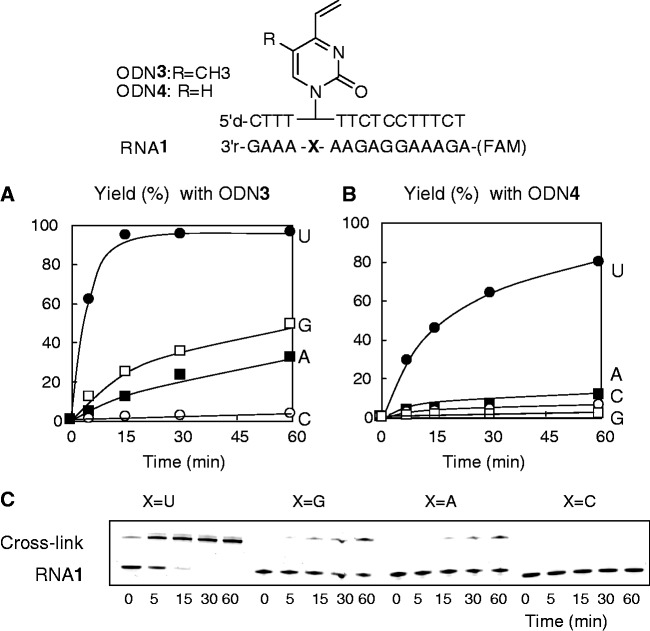
Cross-linking reaction with ODN**3,4** and RNA**1**. Cross-linking conditions: [ODN**3** or **4] = **10 µM, RNA**1** = 1 µM, 50 mM MES buffer, 100 mM NaCl, 37°C, pH 7.0. The reaction was followed by electrophoresis using 15% denatured polyacrylamide gel. The fast- and slow-moving bands represent RNA**1** and the cross-linked products, respectively. The cross-link yield was obtained by quantification of the bands by FAM fluorescence (λ_em_ = 518 nm, λ_ex_ = 494 nm), and it was plotted against time. (**A**) Yield obtained with ODN**3** (R = CH_3_). (**B**) Yield obtained with ODN**4** (R = H). (**C**) An example of gel-shift analysis of the reaction with ODN**3** (R = CH_3_).

**Figure 3.**
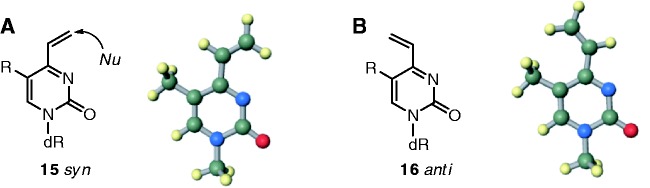
A *syn*- (**15**) and an *anti-*conformation (**16**) of the vinyl-substituted derivative.

The vinyl-ODNs (**3** and **4**) obtained were used for the cross-linking reaction with the target RNA**1** having different nucleoside residues at the complementary positions (Figure 2). Surprisingly, the cross-linking reaction of ODN**3** (R = CH_3_) to the RNA**1** with U at the opposite position (X = U) was complete within 15 min (Figure 2A). The RNA**1** with an opposing G or A (X = G or A) showed lower reactivity, whereas the RNA**1** with an opposing C (X = C) did not form any appreciable cross-link product. Interestingly, ODN**4** (R = H) containing U-vinyl **3** showed much slower reaction rates while retaining the selectivity for U (Figure 2B). Using Arrhenius plots, kinetic parameters for the cross-linking reactions were obtained, which indicated that T-vinyl (**2**) is superior to U-vinyl (**3**), as indicated by the smaller negative value of activation entropy (Supplementary Figure S6 and Supplementary Table S1). It is reasonable to interpret from this result that the vinyl group of the T-vinyl (R = CH_3_) requires less conformational change than that of the U-vinyl (R = H) for cross-link formation to occur. This interpretation is supported by molecular orbital calculations (B3LYP/6-31G*); a *syn*-conformation (**15**), in which the vinyl group is directed towards the Watson–Crick face, is 2.57 kcal/mol more stable than the *anti*-conformation (**16**) of T-vinyl (R = CH_3_), whereas this conformation is only 0.52 kcal/mol more stable in the U-vinyl (R = H) (Figure 3 and Supplementary Figure S6C and D). The 5-methyl group on the T-vinyl (**2**) plays a role in directing the vinyl group to the Watson–Crick face by steric repulsion.

**Figure 4.**
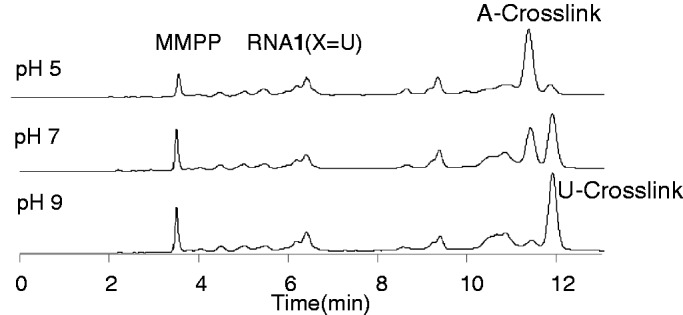
HPLC trace of the cross-linking reaction using ODN**3** and non-labeled RNA**1** (X = U). Cross-linking conditions: [ODN**3**] = 15 µM, RNA**1** = 10 mM, 50 mM MES buffer, 100 mM NaCl, 37°C, pH 5 or pH 7. The reaction at pH9 was performed in 50 mM carbonate buffer. HPLC conditions: column SHISEIDO C18, 4.6 × 250 mm; solvent A 0.1 M TEAA buffer, solvent B CH_3_CN, B 10–20% /20 min 20–100%/25 min, linear gradient; flow rate 1.0 ml/min; UV 254 nm. Each peak was assigned to the A- or U-cross-link, as described in the text. MMPP used for preparation of ODN**3** appeared in HPLC chart.

**Figure 5.**
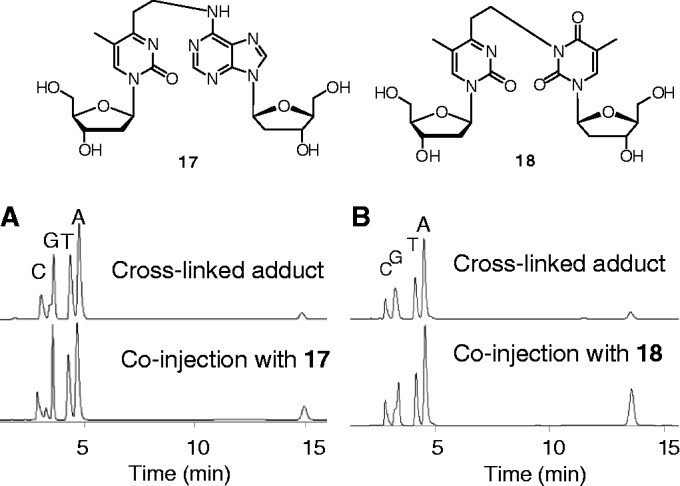
HPLC analysis of the enzymatic hydrolyzates of the cross-linked products obtained at pH 5 (**A**) and those obtained at pH9 (**B**). The peaks appearing at ∼15 min in each chart showed identical retention times and MSs with those corresponding to the authentic samples (**17** and **18**).

Although this new cross-linking agent was designed for reaction with an adenine base, both T–(**2**) and U-vinyl (**3**) showed selectivity for target RNAs having U at the complementary site. To examine the effect of duplex stability on selectivity, *T*_m_ values were measured for each duplex. For this purpose, the vinyl group of ODN**3** (R = CH_3_) was reduced with NaBH_4_ to give a non-reactive ODN that had an ethyl group instead of a vinyl group (Supplementary Figure S4). The *T*_m_ values for the duplexes formed between reduced ODN**3** and RNA**1** (X = U, G, A and C) were determined to be 42.2°C (X = U), 43.5°C (X = A), 49.4°C (X = G) and 41.5°C (X = C), indicating that the cross-linking reaction took place at temperatures lower than the *T*_m_ values, and that the selectivity was not reflective of the *T*_m_ value (Supplementary Figure S5). To gain insight into the cross-linking reaction, it was monitored using HPLC. The reaction mixture at pH 7 gave two major peaks at ∼12 min, both of which were confirmed to be the cross-linked products by Matrix Assisted Laser Desorption Ionization-Time of Flight (MALDI-TOF)/MS (Figure 4 and Supplementary Table S2). The faster peak became predominant after the reaction at pH 5, whereas the slower peak dominated at pH 9. The cross-link peaks were isolated and subjected to enzymatic hydrolysis using bacterial alkaline phosphatase, venom phosphodiesterase and P1 nuclease for the HPLC analysis of the product structure. Interestingly, the faster peak produced an adenosine adduct [electrospray ionization (ESI)-MS *m/e *calcd 520.22 (M + H)^+^, found 520.07]; in contrast, the slower peak gave a uridine adduct (ESI-MS *m/*e calcd 497.19 [M + H]^+^, found 497.30). As DNA substrates showed base selectivity and the pH-dependence of the corresponding HPLC profile similar to those of RNA substrates (Supplementary Figure S7), DNA substrates were used for more detailed analysis. The authentic adducts with dA and T were synthesized by adding tert-butyldimethylsilyl (TBS)-protected dA or T during the synthesis of **9** (Supplementary Schemes S2 and S3). Their structures were confirmed by ^1^H–^1^H correlation spectroscopy for **17** (Supplementary Figure S1) or ^1^H–^13^C hetero-nuclear multiple-bond connectivity for **18** (Supplementary Figure S2). An HPLC comparison with authentic **17** confirmed that the cross-link was predominantly formed with the 6-amino group of dA at acidic pH (Figure 5A). Conversely, Figure 5B clearly shows that the cross-link was formed at the N3 atom of T (**18**) at alkaline pH (Figure 5B). These results suggest that the reactivity of the vinyl group of **2** is affected by the pH of the reaction medium.

#### Determination of the cross-linked products

**Figure 6.**
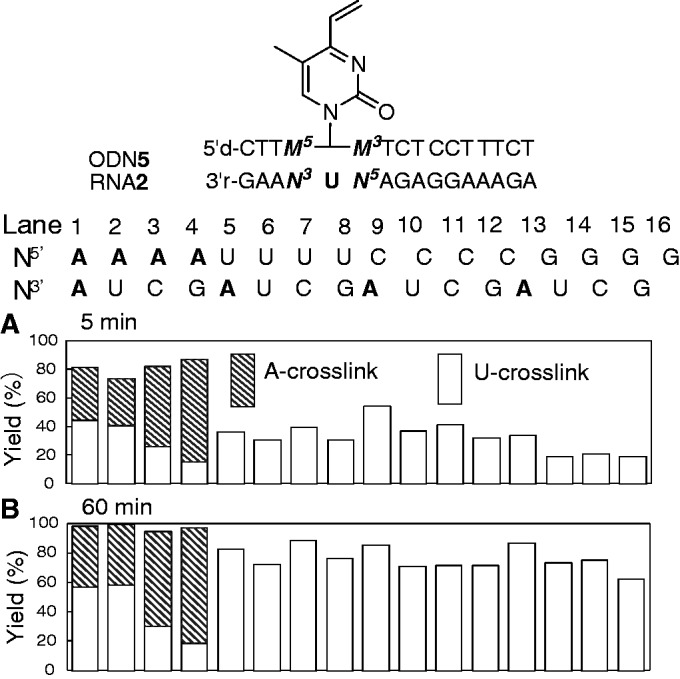
The ratio of A- to U-cross-linking at 5 and 60 min. The ratio was determined using HPLC. The cross-linking conditions were [ODN**5**] = 15 µM, [RNA**2**] = 10 µM, 50 mM MES buffer, 100 mM NaCl, pH 7.0, 37°C. Flanking nucleosides *M*^3^ and *M*^5^ of ODN**5** are complementary to *N*^5^ and *N*^3^, respectively.

As two adenosine residues flank the opposing uridine in the target RNA**1**, it is necessary to determine which of these adenosine residues is responsible for cross-link formation. Accordingly, cross-link formation was analyzed using all 16 possible combinations of RNA**2** (*N^3^-*U-*N^5^*) with various 3′-, 5′-flanking nucleotide relative to U and the complementary ODN**5** (*M*^5^-**2**-*M*^3^) (Figure 6). The yields of the A- and U-adducts were determined using an HPLC analysis of the reaction and the enzymatic hydrolyzates of the isolated products (Supplementary Figure S8). It should be noted that cross-link formation predominantly occurred with an adenine residue at the 5′ position relative to U (striped columns in lanes 1–4). The adenosine residue at the 3′ position did not undergo cross-linking (lanes 5, 9 and 13). Cross-link formation with the opposing U was slower than with A at the 5′-position (white columns in lanes 5–16); however, yields ∼80% were obtained after 60 min (Figure 6B).

**Figure 7.**
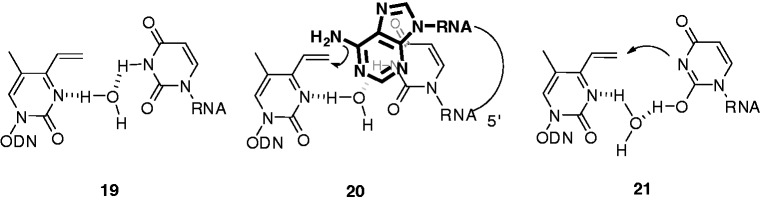
Speculated hydrogen bonds between T-vinyl **2** and a uracil base. A bridging water molecule was assumed in **19**. A close proximity between 6-NH_2_ of 5′-A is shown schematically in **20**. The cross-link formed with the *N*3 of the opposite U likely by the reaction with its enol or enolate form as shown in **21**.

Because having a U residue opposite to T-vinyl **2** is important for efficient cross-link formation with a 5′-A, it may be postulated that this uracil base plays a role in maintaining the duplex structure. As the distance between the T-vinyl derivative and U is too far for direct H-bond formation, we speculate that a bridging water molecule may be important for this H-bonding interaction, as shown in **19** (Figure 7). Such bridging water molecules were shown to contribute to the formation of a U–C base pair in a crystal structure, thus highlighting this interaction (32,33). Molecular modeling (Supplementary Figure S9) indicates that the 6-amino group of the adenine residue at the 5′ side is in van der Waals contact with the vinyl group of **2**, as shown schematically in **20** (Figure 7). The fact that the cross-link reaction was faster at pH 5 might imply that protonation at the N3 atom of **2** increases the reactivity of the vinyl group towards the 6-amino group of 5′-A, which is in close proximity. RNA**2** lacking a 5′-A formed a cross-link with the N3 atom of the opposite U (Figure 6B), most likely by reaction with its enol or enolate form (Figure 7, **21**). Cross-link formation with an opposing U decreased at pH 5 (Figure 4), probably because of low reactivity of its keto form as a major component. The enolate form may also contribute to the selective cross-link formation with an opposing U at pH 9 (Figure 4).

#### Cross-link formation within triplex DNA

**Figure 8.**
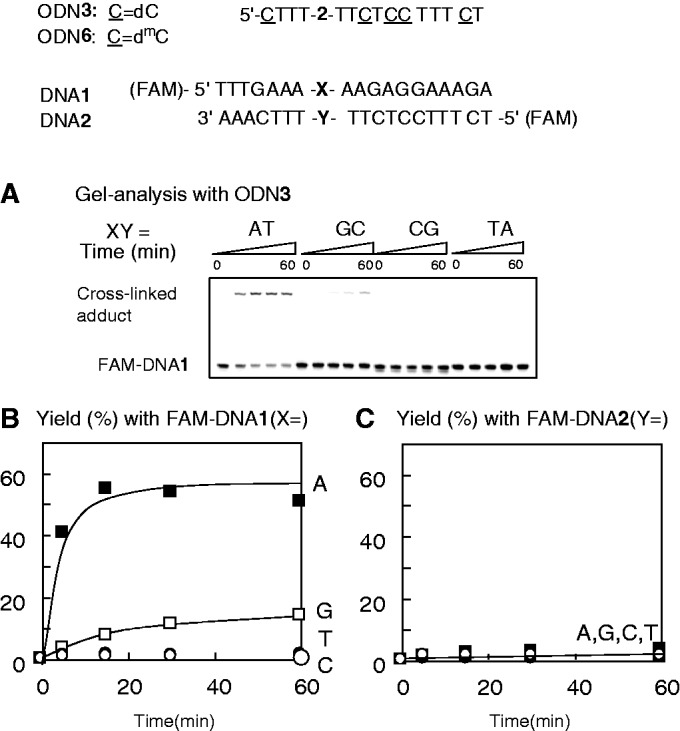
Evaluation of triplex cross-linking. Cross-linking conditions, [ODN**3** or ODN**6**] = 25 µM, [DNA**1**/DNA**2**] = 5 µM, 50 mM MES buffer, 100 mM NaCl, 10 mM MgCl_2_, 25°C, pH 5.0 (C = dC) or pH 6.1 (C = d^m^C). (**A**) Electrophoresis, 15% denatured polyacrylamide gel. The reaction was checked at 0, 5, 15, 30 and 60 min. (**B**) The yield of the cross-linked product was obtained using FAM-labeled DNA**1**. (**C**) The yield was obtained using FAM-labeled DNA**2**.

**Figure 9.**
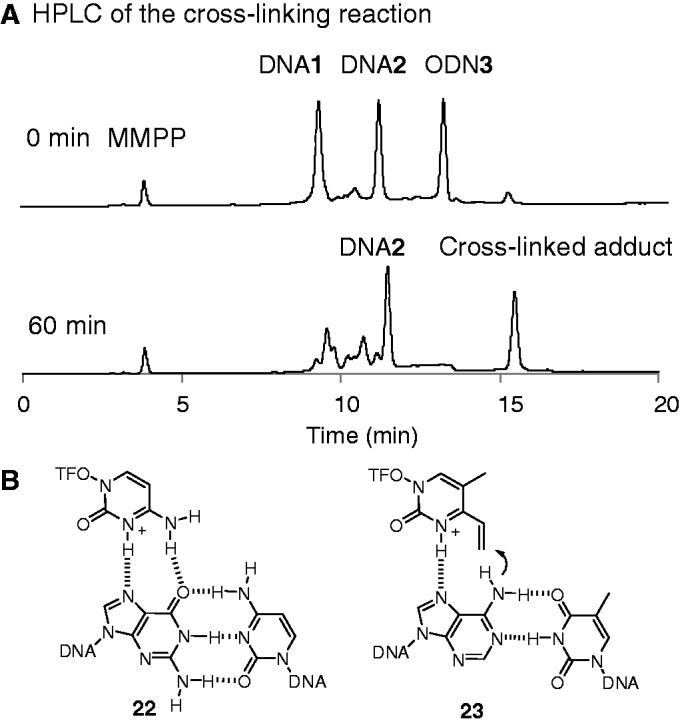
(**A**) HPLC analysis of cross-link formation in the triplex DNA. The reaction was done using ODN**3** (30 μM) and DNA**1** and **2** (10 μM each) in 50 mM MES buffer (pH 5.0), including 100 mM NaCl and 10 mM MgCl_2_ at 25°C for 1 h (**B**) Expected complex structures in the triplex DNA between a protonated C and a GC pair (**22**) and that between a protonated **2** and an AT pair (**23**). MMPP used for preparation of ODN3 appeared in HPLC chart.

We next investigated cross-link formation in triplex DNA. ODN**3** contains T-vinyl **2** in the homopyrimidine strand and can, therefore, form triplex DNA with a homopurine–homopyrimidine duplex. ODN**6** contains 5-methylcytidine (d^m^C) instead of dC, which is used for triplex formation under weakly acidic conditions. The target duplex was prepared using DNA**1** and DNA**2**, either of which was labeled with 6-carboxyfluorescein-aminohexyl (FAM) to clarify the cross-linked strand. Figure 7A illustrates gel analysis of the reaction between ODN**3** and the duplex formed with FAM-labeled DNA**1** and non-labeled DNA**2**. Slow mobility bands were formed only in the reaction with the duplex containing XY = AT. Efficiency and selectivity were clearly demonstrated by examining the time course of the reaction (Figure 8B). When the duplex was formed with non-labeled DNA**1** and FAM-labeled DNA**2**, no slow mobility bands were observed (Figure 8C). In addition, FAM–DNA**1** alone did not produce the slow mobility bands, clearly indicating that cross-link formation took place with DNA**1** in the triplex. This was also confirmed by an HPLC analysis of the cross-linking reaction medium using non-labeled DNA**1** and DNA**2**. The ODN**3** and DNA**1** peaks disappeared, whereas the DNA**2** peak remained and the cross-linked product appeared at ∼15 min in the HPLC profile (Figure 9A). As the reaction was performed at acidic conditions, the hydration reaction of the vinyl group of ODN**3** took place rapidly to produce side products observed between the peaks of DNA**1** and **2**. The cross-linked product was subjected to enzymatic hydrolysis and HPLC analysis to demonstrate that the cross-link was formed with the 6-amino group of adenine (Figure 5, **17**). ODN**6**, containing d^m^C instead of dC, produced the cross-link at weakly acidic pH, whereas no adduct was formed with ODN**3** under the same conditions (Supplementary Figure S10). As a protonated dC is responsible for the formation of triplex DNA containing CGC base triplets (Figure 9, **22**), and d^m^C is protonated at weakly acidic pH, these results also support that cross-link formation takes place in the triplex DNA. The reactivity of the vinyl group is enhanced at acidic pH because of protonation at N3; therefore, such a protonated form might contribute to the access of the vinyl group to 6-NH_2_ of the adenosine residue in the homopurine strand as depicted in **23** (Figure 9B).

## CONCLUSION

Analogues of thymidine (T-vinyl, **2**) and 2′-deoxyuridine (U-vinyl, **3**) containing 4-vinyl-sustituents were designed as new cross-linking agents. In these compounds, the vinyl group is oriented towards the Watson–Crick face to facilitate a reaction with the amino group of the adenine base. ODN containing **2** or **3** exhibited rapid cross-link formation with RNA substrates containing U at the position opposite to **2** or **3**. In the case of RNA**1** (X = U) containing A at the 5′ side of U, a cross-link formed with the 6-amino group of the 5′-A. RNA**1** (X = U) lacking 5′-A formed a cross-link with the N3 atom of a U at the opposing position. T-vinyl (**2**) showed more efficient reactivity than U-vinyl (**3**), most likely because the direction of the vinyl group of T-vinyl is pre-organized towards the Watson–Crick face by the steric bulkiness of the 5-methyl group. The ODN probe incorporating **2** efficiently formed interstrand cross-links in parallel-type triplex DNA with high selectivity for dA in the homopurine strand. Thus, the efficiency of the cross-linking reaction of **2** has been demonstrated for DNA/RNA, DNA/DNA duplexes and triplex DNA. The efficiency and selectivity suggest that T-vinyl **2** may become a unique tool in biological and materials research.

## SUPPLEMENTARY DATA

Supplementary Data are available at NAR Online: Supplementary Tables 1 and 2, Supplementary Figures 1–10 and Supplementary Schemes 1–3.

## FUNDING

Grant-in-Aid for Scientific Research (S) [21229002] from the Japan Society for the Promotion of Science (JSPS); CREST from the Japan Science and Technology Agency. Funding for open access charge: Grant-in-Aid for Scientific Research (S) [21229002] from the Japan Society for the Promotion of Science (JSPS).

*Conflict of interest statement.* None declared.

## Supplementary Material

Supplementary Data
