# Supplementary material for: 4-vinyl-substituted pyrimidine nucleosides exhibit the efficient and selective formation of interstrand cross-links with RNA and duplex DNA
Source: Nucleic Acids Res. 2013 Jun 18;41(13):6774–81. doi: 10.1093/nar/gkt197 (PMC3711451; doi:10.1093/nar/gkt197)
Supplement: Supplementary Data [file supp_41_13_6774__index.html]

4-vinyl-substituted pyrimidine nucleosides exhibit the efficient and selective formation of interstrand cross-links with RNA and duplex DNA — 4-vinyl-substituted pyrimidine nucleosides exhibit the efficient and selective formation of interstrand cross-links with RNA and duplex DNA — Supplementary Data 

# 4-vinyl-substituted pyrimidine nucleosides exhibit the efficient and selective formation of interstrand cross-links with RNA and duplex DNA

## Supplementary Data

files

**Files in this Data Supplement:**

- Supplementary Data - pdf file
