# Supplementary material for: 4-vinyl-substituted pyrimidine nucleosides exhibit the efficient and selective formation of interstrand cross-links with RNA and duplex DNA
Source: Nucleic Acids Res. 2013 Jun 18;41(13):6774–81. doi: 10.1093/nar/gkt197 (PMC3711451; doi:10.1093/nar/gkt197)
Supplement: Supplementary Data [file supp_gkt197_nar-00232-f-2013-File012.pdf]

Supplementary Data

4-Vinyl-Substituted Pyrimidine Nucleosides Exhibit the Efficient and Selective Formation of Interstrand Cross-Links with RNA and duplex DNA

Atsushi Nishimoto,1 Daichi Jitsuzaki,1 Kazumitsu Onizuka,1 Yosuke Taniguchi,1,3 Fumi Nagatsugi,2,3 Shigeki Sasaki1,3

[1]Graduate School of Pharmaceutical Sciences, Kyushu University, Fukuoka 812-8582 Japan,
[2]Institute of Multidisciplinary Research for Advanced Materials Tohoku University, Sendai 980-8577, Japan,
[3]CREST, Japan Science and Technology Agency, Saitama 332-0012, Japan.

E-mail: sasaki@phar.kyushu-u.ac.jp

Synthesis of **13** and **14**. Scheme S1 2
Synthesis of the authentic adduct **17**. Scheme S2 and Figure S1 9
Synthesis of the authentic adduct **18**. Scheme S3 and Figure S2 11
Synthesis of **ODN3** and Figure S3 14
Reduction of **ODN3** and Figure S4 and S5 15
The cross-linking reaction using the RNA and DNA substrate 16
The Arrhenius plots of the cross-linking reaction, Figure S6 and Table S1 17
The cross-linking data for the DNA substrate and Figure S7 18
HPLC chart for obtaining the data shown in Figure 4, Figure S8 19
The enzymatic digestion 19
Molecular Modeling in Figure S9 20
The triplex cross-link formation at different pH in Figure S10 21
Table S2. MALDI-TOF/MS data 22

MATERIALS AND METHODS

Scheme S1

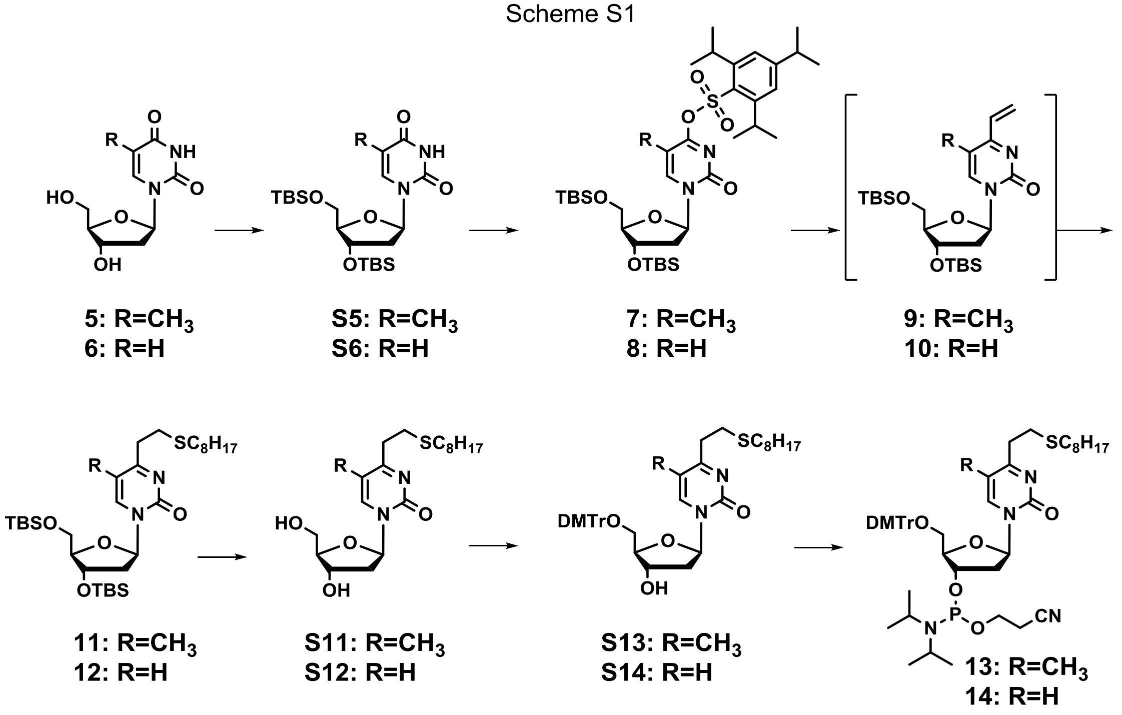

3', 5'-Bis-*O*-(tert-butyldimethylsilyl)-4-*O*-2,4,6-triisopropylbenzenesulfonylthymidine (7). Dry Et_3N (0.59 mL, 4.25 mmol), 2,4,6-triisopropylbenzenesulfonyl chloride (642 mg, 2.12 mmol) and DMAP (26 mg, 0.21 mmol) were added to a solution of 3', 5'-di-*O*-tert-butyldimethylsilylthymidine (**S5**, 0.5 g, 1.06 mmol) in dry CH_2Cl_2 (5 mL) at 0°C under an argon atmosphere. After 43 h at room temperature, the reaction mixture was diluted with $CHCl_3$ (15 mL), then washed with H_2O and brine. The organic layer was dried over Na_2SO_4, filtered, and concentrated under reduced pressure. The residue was purified by silica gel chromatography (hexane-ethyl acetate = 30:1, v/v) to give **7** as a colorless solid (0.73 g, 0.98 mmol, 93%): mp 153.2-153.5 ^{o}C; ^{1}H-NMR (400 MHz, $CDCl_{3)}$ δ (ppm) 7.95 (1H, s), 7.17 (2H, s), 6.10 (1H, t, *J* = 6.1 Hz), 4.33- 4.26 (3H, m), 3.94 (1H, dt, *J* = 3.1,

2.4 Hz), 3.87 (1H, dd, *J* = 11.6, 2.4 Hz), 3.72 (1H, dd, *J* = 11.6, 2.4 Hz), 2.88 (1H, quintet, *J* = 7.0 Hz), 2.46 (1H, ddd, *J*= 13.4, 6.1, 3.7 Hz), 2.01 (3H, s), 1.94 (1H, ddd, *J* = 13.4, 6.7, 6.1 Hz), 1.29 (6H, d, *J* = 7.0 Hz), 1.24 (6H, t, *J* = 6.1 Hz), 1.23 (6H, t, *J* = 7.0 Hz), 0.88 (9H, s), 0.84 (9H, s), 0.07 (3H, s), 0.06 (3H, s), 0.03 (3H, s), 0.02 (3H, s); ^{13}C-NMR (125 MHz, $CDCl_3$) δ (ppm) 166.2, 154.2, 153.7, 151.1, 143.0, 131.0, 124.0, 103.3, 88.4, 87.3, 71.6, 62.5, 42.4, 34.2, 29.6, 25.9, 25.7, 24.6, 24.3, 23.4, 18.4, 18.0, 12.4, -4.6, -4.9, -5.4; IR (cm^{-1}) 2955, 2349, 1685, 1537; HR ESI MS (m/z) calcd for $C_{37}H_{65}N_2O_7SSi_2$ $[M+H]^+$ 737.4046, found 737.4055.

4-(2-Octylthioethyl)-1-[(1*R*,3*S*,4*R*)-2-deoxy-3,5-bis-*O*-(tert-butyldimethylsilyl)tetrahydrofuran-1-yl]-5-methyl-2(1*H*)-pyrimidinone (11).

$Pd(PPh_3)_4$ (312 mg, 0.27 mmol), LiBr (283 mg, 3.26 mmol) , K_2CO_3 (225 mg, 1.63 mmol) and 2,4,6-trivinylcyclotriboroxane pyridine complex (915 mg, 3.80 mmol) were added to a solution of **7** (2 g, 2.71 mmol) in 1,4-dioxane (60 mL) and H_2O (20 mL) at 120 ^oC under an argon atmosphere. After 45 min, CH_3CN (40 mL) and $C_8H_{17}SH$ (0.71 ml, 4.07 mmol) were added to the reaction mixture at room temperature, and then the mixture was stirred for an additional 30 min at the same temperature. The reaction mixture was diluted with $CHCl_3$ (80 mL), then washed with H_2O and brine. The organic layer was dried over Na_2SO_4, filtered, and concentrated under reduced pressure. The residue was purified by silica gel chromatography ($CHCl_3$-MeOH = 1:0 to 49:1, v/v) to give **11** as a yellow oil (1.60 g, 2.55 mmol, 94% 2 steps): ^{1}H-NMR (400 MHz, $CDCl_3$) δ(ppm) 7.91 (1H, s), 6.23 (1H, t, *J* = 6.4 Hz), 4.35 (1H, dt, *J* = 6.4, 3.7 Hz), 3.98 (1H, dt, *J* = 3.4, 2.8 Hz), 3.90 (1H, dd, *J* = 11.3, 2.8 Hz), 3.75 (1H, dd, *J* = 11.3, 2.8 Hz), 2.97-2.89 (4H, m), 2.60-2.53 (1H, m), 2.55 (2H, t, *J*=

7.6 Hz), 2.06 (3H, s), 2.01 (1H, ddd, *J* = 13.4, 7.0, 6.4 Hz), 1.57 (2H, t, *J* = 7.6 Hz), 1.34-1.25 (10H, broad), 0.89-0.84 (3H, m), 0.89 (9H, s), 0.87 (9H, s) 0.09 (3H, s), 0.08 (3H, s), 0.05 (3H, s), 0.04 (3H, s); ^{13}C-NMR (125 MHz, $CDCl_3$) δ(ppm) 176.7, 155.3, 140.0, 111.6, 88.3, 87.0, 71.5, 62.5, 42.4, 36.0, 32.6, 31.8, 29.7, 29.2, 29.2, 29.0, 28.9, 25.9, 25.7, 22.6, 18.4, 18.0, 14.9, 14.9, 14.0, -4.6, -4.9, -5.4; IR (cm^{-1}): 2929, 1665, 1509, 1463; HR ESI MS (m/z) calcd for $C_{32}H_{63}N_2O_4SSi_2$ $[M+H]^+$ 627.4042, found 627.4062.

4-(2-Octylthioethyl)-1-[(1*R*,3*S*,4*R*)-2-deoxy-3-*O*-[2-cyanoethyl *N*,*N*-bis(1-methylethyl)phosphoramidite]-5-*O*-[bis(4-methoxyphenyl)phenylmethyl]tetrahydrofuran-1-yl]-5-methyl-2(1*H*)-pyrimidinone (13). A solution of nBu_4NF in THF (1.0 M solution, 3.11 ml, 3.11 mmol) was added to a solution of **11** (930 mg, 1.48 mmol) in dry THF (15 mL) at room temperature under an argon atmosphere. After 20 min, the reaction mixture was concentrated under reduced pressure. The residue was purified by silica gel chromatography ($CHCl_3$-MeOH = 1:0 to 20:1 to 10:1, v/v) to give **S11** as a yellow foam (466 mg, 1.17 mmol, 79%): ^{1}H-NMR (500 MHz, $CDCl_3$) δ(ppm) 8.12 (1H, s), 6.09 (1H, t, *J* = 5.7 Hz), 4.54 (1H, dt, *J* = 5.7, 5.2 Hz), 4.03 (1H, dt, *J* = 5.2, 2.7 Hz), 3.92 (1H, dd, *J* = 12.6, 2.7 Hz), 3.90 (1H, dd, *J* = 12.6, 2.5 Hz), 2.86 (2H, t, *J* = 6.0 Hz), 2.56-2.51 (1H, m), 2.54 (2H, dd, *J*= 7.6, 7.3 Hz), 2.31 (1H, dt, *J* = 13.1, 6.2, 5.7 Hz), 2.08 (3H, s), 1.58-1.53 (2H, m), 1.41-1.30 (2H, broad), 1.29-1.24 (10H, broad), 0.85 (3H, t, *J* = 6.9 Hz); ^{13}C-NMR (125 MHz, $CDCl_3$) δ(ppm) 177.2, 155.5, 142.0, 112.9, 88.1, 87.8, 69.6, 61.3, 40.9, 35.9, 32.6, 31.8, 29.7, 29.2, 29.2, 29.1, 28.9, 22.6, 14.9, 14.1; IR (cm^{-1}) 3343, 2925, 1654, 1615, 1514; HR ESI MS (m/z) calcd for $C_{20}H_{35}N_2O_4S$ $[M+H]^+$ 399.2312, found 399.2299.

Thioanisole (324 μL, 2.76 mmol), diisopropylethylamine (480 μL, 2.76 mmol) and dimethoxytrityl chloride (140 mg, 0.41 mmol) were added to a solution of **S11** (110 mg, 0.28 mmol) in dry CH_2Cl_2 (2 mL) at 0 ^oC under an argon atmosphere. After being stirred for 45 min, the reaction mixture was diluted with ethyl acetate (20 mL), and washed with H_2O and brine. The organic layer was dried over Na_2SO_4, filtered, and concentrated under reduced pressure. The residue was purified by silica gel chromatography ($CHCl_3$-MeOH = 1:0 to 50:1 to 5:1, v/v) to give **S13** as a yellow foam (170 mg, 0.24 mmol, 88%): H-NMR (400 MHz, $CDCl_{3)}$ δ(ppm) 8.00 (1 H, d, *J* = 0.6 Hz), 7.36 (2H, dd, *J* = 7.0, 1.5 Hz), 7.28-7.24 (6H, m), 7.21 (1H, dt, *J* = 7.0, 1.5 Hz), 6.81 (4H, d, *J* = 8.9 Hz), 6.30 (1H, t, *J* = 6.1 Hz), 4.54 (1H, dt, *J* = 6.1, 3.7 Hz), 4.15 (1H, dt, *J* = 3.6, 3.6 Hz), 3.77 (6H, s), 3.47 (1H, dd, *J* = 10.7, 3.4 Hz), 3.35 (1H, dd, *J* = 10.7, 3.4 Hz), 2.93-2.88 (2H, m), 2.84-2.79 (2H, m), 2.68 (1H, ddd, *J*= 13.7, 6.1, 4.3 Hz), 2.53 (2H, t, *J*= 7.6 Hz), 2.26 (1H, ddd, *J* = 13.7, 6.4, 6.1 Hz), 1.67 (3H, d, *J* = 0.6 Hz), 1.61-1.53 (2H, m), 1.36-1.25 (10H, broad), 0.85 (3H, t, *J* = 7.0 Hz); ^{13}C-NMR (125 MHz, $CDCl_3$) δ(ppm) 177.0, 158.7, 155.4, 144.4, 140.3, 135.5, 130.0, 130.0, 128.1, 127.9, 127.0, 113.3, 112.4, 87.2, 86.8, 86.5, 71.7, 63.2, 55.2, 42.2, 36.0, 32.6, 31.8, 29.7, 29.2, 29.0, 28.9, 22.6, 14.3, 14.1; IR (cm^{-1}): 3371, 2928, 1655, 1608, 1509; HR ESI MS (m/z): calcd for $C_{41}H_{53}N_2O_6S$ $[M+H]^+$ 701.3619, found 701.3658.

Diisopropylethylamine (450 μL, 2.57 mmol) and 2-cyanoethyl *N*,*N*-diisopropylchlorophosphoramidite (286 μL, 1.28 mmol) were added to the solution of **S13** (300 mg, 0.43 mmol) in dry CH_2Cl_2 (4 mL) at 0 ^oC under an argon atmosphere. After being stirred for 1 h, the reaction mixture was quenched with saturated aqueous $NaHCO_3$ (20 mL), extracted with ethyl acetate (20 mL×3). The organic layer was dried over Na_2SO_4,

filtered, and concentrated under reduced pressure. The residue was purified by silica gel chromatography (hexane-ethyl acetate = 3:1 to 2:1, v/v) to give the material, which was crystallized in hexane at -78 °C. The hexane was removed by decantation, and the solid material was dried in a vacuum for several hours to give **13** as a white foam (279 mg, 0.31 mmol, 72%): ^{1}H-NMR (400 MHz, $CDCl_{3)}$ δ(ppm) 8.03 (0.5H, s), 7.97 (0.5H, s), 7.38-7.36 (2H, m), 7.31-7.25 (7H, m), 6.80 (5H, ddd, *J* = 8.9, 5.5, 2.1 Hz), 6.31 (0.5H, t, *J* = 6.4 Hz), 6.27 (0.5H, t, *J* = 6.1 Hz), 4.63-4.55 (1H, m), 4.18 (1H, dt, *J* = 6.7, 3.7 Hz), 3.78 (3H, s), 3.77 (3H, s), 3.75-3.69 (1H, m), 3.59-3.48 (4H, m), 3.31 (1H, dt, *J* = 11.3, 3.1 Hz), 2.92 (2H, dd, *J* = 7.9, 7.0 Hz), 2.80 (2H, dd, *J* = 8.6, 7.6 Hz), 2.77-2.68 (1H, m), 2.59 (1H, t, *J*= 6.4 Hz), 2.53 (2H, t, *J* = 7.6 Hz), 2.37 (1H, t, *J*= 6.4 Hz), 2.34-2.24 (1H, m), 1.59 (1.5H, s,), 1.57 (1.5H, s,), 1.61-1.53 (2H, m), 1.36-1.18 (10H, broad), 1.14 (9H, dd, *J* = 6.7, 4.0 Hz), 1.02 (3H, d, *J* = 7.0 Hz), 0.86 (3H, t, *J* = 6.7 Hz); ^{13}C-NMR (125 MHz, $CDCl_{3})$ δ (ppm) 176.9, 175.6, 158.7, 154.9, 144.3, 140.1, 135.4, 130.2, 130.1, 128.3, 128.2, 127.9, 127.1, 117.5, 113.2, 112.2, 86.9, 85.7, 73.2, 73.0, 62.8, 62.4, 58.2, 55.3, 55.2, 43.4, 43.3, 43.3, 43.2, 41.2, 40.9, 36.0, 32.6, 32.0, 31.8, 29.7, 29.2, 29.2, 28.9, 28.9, 24.6, 24.5, 22.6, 20.4, 14.2, 14.1; ^{31}P-NMR (161 MHz, CD $Cl_{3)}$ δ(ppm) 149.3, 148.6; IR (cm^{-1}): 2964, 2927, 1663, 1608, 1509; HR ESI MS (m/z): calcd for $C_{50}H_{70}N_{4}O_{7}PS$ $[M+H]^{+}$ 901.4697, found 901.4686.

4-(2-Octylthioethyl)-1-[(1*R*,3*S*,4*R*)-2-deoxy-3-*O*-[2-cyanoethyl *N*,*N*-bis(1-methylethyl)phosphoramidite]-5-*O*-[bis(4-methoxyphenyl)phenylmethyl]tetrahydrofuran-1-yl]-2(1*H*)-pyrimidinone (14). The 2'-deoxyuridine derivative (**14**) was synthesized by a similar procedure as described for the synthesis of **13** from

2'-deoxyuridine (**6**).

8: ^{1}H-NMR (400 MHz, $CDCl_{3)}$ δ(ppm) 8.43 (1H, d, *J* = 7.3 Hz), 7.18 (2H, s), 6.06 (1H, dd, *J* = 6.4, 4.3 Hz), 5.99 (1H, d, *J* = 7.4 Hz), 4.31 (1H, dt, *J* = 6.1, 5.2 Hz), 4.24 (2H, dq, *J* = 6.7 Hz), 3.92 (1H, dd, *J* = 12.2, 2.4 Hz), 3.91-3.89 (1H, m), 3.74 (1H, dd, *J* = 12.2, 2.5 Hz), 2.88 (1H, dq, *J* = 6.9 Hz), 2.46 (1H, ddd, *J* = 13.4, 6.4, 6.4 Hz), 2.09 (1H, ddd, *J* = 13.4, 6.4, 4.3 Hz), 1.29 (6H, d, *J* = 6.7 Hz), 1.25 (6H, d, *J* = 5.2 Hz), 1.23 (6H, d, *J* = 5.5 Hz), 0.89 (9H, s), 0.84 (9H, s), 0.08 (3H, s), 0.07 (3H, s), 0.02 (6H, s); ^{13}C-NMR (125 MHz, $CDCl_3$) δ (ppm) 167.0, 154.4, 153.9, 151.9, 146.0, 130.8, 124.0, 94.5, 87.9, 87.3, 69.7, 61.6, 42.2, 34.3, 29.7, 25.9, 25.7, 24.6, 24.4, 23.5, 18.3, 17.9, -4.5, -5.0, -5.5; IR (cm^{-1}) 2956, 2930, 2858, 1686, 1543, 1461,1256; HR ESI MS (m/z) calcd for $C_{36}H_{63}N_2O_7SSi_2$ $[M+H]^+$ 723.3889, found 723.3934.

12: ^{1}H-NMR (400 MHz, $CDCl_{3)}$ δ(ppm) 8.32 (1H, d, *J* = 7.0 Hz), 6.18 (1H, dd, *J* = 6.4, 4.3 Hz), 6.16 (1H, d, *J* = 6.7 Hz), 4.34 (1H, dt, *J* = 6.4, 4.9 Hz), 3.95-3.92 (2H, m), 3.75 (1H, dd, *J* = 12.2, 2.7 Hz), 2.90-2.82 (4H, m), 2.53 (1H, ddd, *J* = 13.4, 6.4, 6.4 Hz), 2.51 (2H, t, *J* = 7.5 Hz), 2.12 (1H, ddd, *J* = 13.4, 6.4, 4.6 Hz), 1.55 (2H, q, *J* = 14.8, 7.6 Hz), 1.34-1.33 (2H, m), 1.24 (8H, s), 1.89 (9H, s), 0.87-0.83 (12H, m), 0.08 (3H, s), 0.07 (3H, s), 0.03 (3H, s), 0.02 (3H, s); ^{13}C-NMR (125 MHz, $CDCl_3$) δ (ppm) 177.2, 155.4, 142.6, 103.9, 87.8, 87.0, 69.8, 61.7, 42.2, 38.7, 32.4, 31.8, 29.6, 29.2, 29.1, 28.9, 25.9, 25.7, 22.6, 18.3, 17.9, 14.0, -4.6, -5.0, -5.5, -5.6; IR (cm^{-1}): 2953, 2928, 2856, 1667, 1522, 1463, 1254; HR ESI MS (m/z) calcd for $C_{31}H_{61}N_2O_4SSi_2$ $[M+H]^+$ 613.3885, found 613.3856.

S12: ^{1}H-NMR (500 MHz, $CDCl_{3)}$ d (ppm) 8.51 (1H, d, *J* = 6.9 Hz), 6.45 (1H, d, *J* = 6.9 Hz), 6.08 (1H, dd, *J* = 4.4, 2.1 Hz), 4.49 (1H, dt, *J* = 6.0 Hz), 4.46 (1H, s), 4.12 (1H, s),

4.02-4.00 (1H, m), 3.93 (2H, s), 2.85-2.83 (4H, m), 2.57 (1H, ddd, *J* = 13.5, 6.4, 6.4 Hz), 2.51 (2H, t, *J* = 7.4 Hz), 2.27 (1H, ddd, *J* = 13.5, 6.4, 4.4 Hz), 1.55 (2H, q, *J* = 14.5, 7.6 Hz), 1.37-1.31 (2H, m), 1.29-1.21 (8H, m), 0.85 (3H, t, *J* = 7.0 Hz); ^{13}C-NMR (125 MHz, $CDCl_3$) d (ppm) 177.2, 155.4, 142.6, 103.9, 87.8, 87.0, 69.8, 61.7, 42.2, 38.7, 32.4, 31.8, 29.6, 177.8, 155.7, 144.1, 104.9, 87.8, 87.8, 68.7, 60.7, 40.9, 38.4, 32.3, 31.8, 29.7, 29.6, 29.2, 28.9, 22.6, 14.1; IR (cm^{-1}) 3387, 2924, 2854, 1646, 1527, 1459, 1275; HR ESI MS (m/z) calcd for $C_{19}H_{33}N_2O_4S$ [M+H]$^+$ 385.2156, found 385.2186.

S14: ^{1}H-NMR (400 MHz, $CDCl_3$) d (ppm) 8.25 (1H, d, *J* = 6.7 Hz), 7.36 (2H, d, *J* = 7.0 Hz), 7.29-7.21 (7H, m), 6.81 (4H, d, *J* = 8.9 Hz), 6.20 (1H, t, *J* = 5.8 Hz), 5.95 (1H, d, *J* = 6.7 Hz), 4.51 (1H, dt, *J* = 5.8, 5.2 Hz), 4.11 (1H, dt, *J* = 4.5, 3.4 Hz), 3.77 (6H, s), 3.49 (1H, dd, *J* = 10.8, 3.4 Hz), 3.41 (1H, dd, *J* = 10.8, 3.4 Hz), 2.88-2.79 (4H, m), 2.71 (1H, ddd, *J* = 13.7, 5.8, 5.8 Hz), 2.51 (2H, t, *J* = 7.5 Hz), 2.27 (1H, ddd, *J* = 13.7, 5.8, 5.8 Hz), 1.56 (2H, q, *J* = 14.8, 7.6 Hz), 1.36-1.32 (2H, m), 1.25 (8H, s), 0.86 (3H, t, *J* = 6.9 Hz); IR (cm^{-1}): 3351, 2927, 2853, 1647, 1609, 1526, 1509, 1461, 1250; HR ESI MS (m/z) calcd for $C_{40}H_{51}N_2O_6S$ [M+H]$^+$ 687.3462, found 687.4383.

14: ^{1}H-NMR (400 MHz, $CDCl_3$) d (ppm) 8.29 (0.5H, d, *J* = 7.0 Hz), 8.21 (0.5H, d, *J* = 7.0 Hz), 7.38-7.35 (2H, m), 7.23-7.20 (7H, m), 6.82 (2H, d, *J* = 5.5 Hz), 6.80 (2H, d, *J* = 5.5 Hz), 6.22 (0.5H, t, *J* = 6.8 Hz), 6.20 (0.5H, t, *J* = 6.7 Hz), 5.87 (0.5H, d, *J* = 7.0 Hz), 5.86 (0.5H, d, *J* = 6.7 Hz), 4.65-4.56 (1H, m), 4.18-4.16 (1H, m), 3.78 (3H, s), 3.77 (3H, s), 3.75-3.69 (1H, m), 3.62-3.50 (4H, m), 3.37 (1H, dt, *J* = 11,3.4 Hz), 2.86-2.81 (4H, m), 2.78-2.71 (1H, m), 2.59 (1H, t, *J* = 6.3 Hz), 2.51 (2H, t, *J* = 7.5 Hz), 2.41 (1H, t, *J* = 6.4 Hz), 2.35-2.25 (1H, m), 1.56 (2H, q, *J* = 14.8, 7.5 Hz), 1.36-1.32 (2H, m), 1.28-1.20 (8H, m),

1.15 (3H, d, J = 5.5 Hz), 1.14 (3H, d, J = 5.8 Hz), 1.04 (3H, d, J = 6.7 Hz), 0.87 (3H, d, J = 6.7 Hz), 0.86 (3H, t, J = 6.7 Hz); ^{31}P-NMR (161 MHz, $CDCl_3$) d (ppm) 149.5, 148.9; IR (cm^{-1}): 2964, 2928, 2855, 1663, 1609, 1522, 1509, 1463, 1251; HR ESI MS (m/z) calcd for $C_{49}H_{68}N_4O_7PS$ $[M+H]^+$ 887.4541, found 887.4573.

Scheme S2. Synthesis of the authentic adenosine adduct

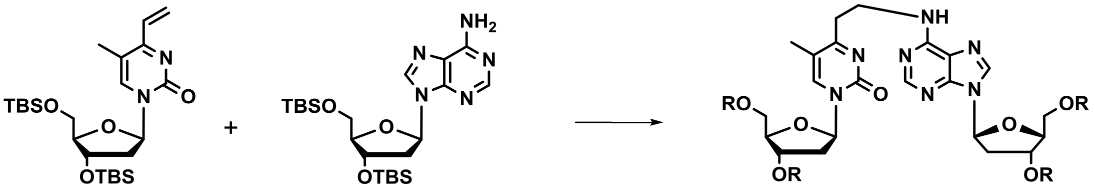

9 3',5'-*O*-diTBS-2'-deoxyadenosine **S17: R=TBS** **17: R=H**

Synthesis of the authentic adduct with 2'-deoxyadenosine (17)

$Pd(PPh_3)_4$ (78 mg, 0.07 mmol), LiBr (70 mg, 0.81 mmol) , K_2CO_3 (56 mg, 0.41 mmol) and 2,4,6-Trivinylcyclotriboroxane pyridine complex (228 mg, 0.95 mmol) were added to a solution of **7** (500 mg, 0.69 mmol) in 1,4-dioxane (15 mL) and H_2O (5 mL) at 120 ^oC under an argon atmosphere, and the mixture was stirred for 1 h. The vinyl compound **9** was synthesized in the mixture, to which a solution of 3',5'-*O*-di(TBS)-2'-deoxyadenosine (325 mg, 0.68 mmol) in CH_3CN (20 mL) was added at room temperature, and then the mixture was stirred for an additional 2 days at 40 ^oC. The reaction mixture was diluted with $CHCl_3$ (80 mL), then washed with H_2O and brine. The organic layer was dried over Na_2SO_4, filtered, and concentrated under reduced pressure. The residue was purified by silica gel chromatography ($CHCl_3$-MeOH = 1:0 to 100:1 to 50:1 to 20:1 to 10:1, v/v) and preparative thin-layer chromatography ($CHCl_3$-MeOH = 10:1, v/v) to give **S17** as a yellow foam (120

mg, 0.12 mmol, 18% 2 steps): ^{1}H-NMR (400 MHz, $CDCl_3$) d (ppm) 8.33 (1H, s), 8.00 (1H, s), 7.85 (1H, s), 6.53 (1H, broad), 6.40 (1H, t, J = 6.4 Hz), 6.23 (1H, t, J = 6.1 Hz), 4.57 (1H, dt, J = 5.5, 3.4 Hz), 4.34 (1H, dt, J = 6.1, 3.7 Hz), 4.14 (2H, broad), 3.97-3.95 (2H, m), 3.89 (1H, dt, J = 11.3, 2.5 Hz), 3.81 (1H, dt, J = 11.3, 4.6 Hz), 3.75 (1H, dt, J = 11.6, 3.7 Hz), 3.73 (1H, dt, J = 11.3, 3.7 Hz), 2.97 (2H, t, J = 6.1 Hz), 2.62-2.52 (2H, m), 2.37 (1H, ddd, J = 13.1, 6.1, 3.7 Hz), 2.05-1.98 (1H, m), 2.00 (3H, s), 0.88 (18H, s), 0.87 (9H, s), 0.87 (9H, s), 0.07 (12H, s), 0.05 (9H, s), 0.04 (3H, s); ^{13}C-NMR (125 MHz, $CDCl_3$) d (ppm) 176.1, 155.2, 154.7, 152.9, 148.7, 139.9, 138.1, 120.2, 112.2, 88.2, 87.8, 87.0, 84.1, 71.9, 71.4, 62.8, 62.5, 42.4, 41.1, 36.9, 35.4, 25.9, 25.9, 25.7, 18.4, 18.4, 18.0, 14.7, -4.5, -4.7, -4.8, -4.9, -5.4, -5.5; IR (cm^{-1}): 3285, 2954, 2929, 2857, 1665, 1619, 1579, 1509, 1472; HR ESI MS (m/z) calcd for $C_{46}H_{86}N_7O_7Si_4$ $[M+H]^+$ 960.5660, found 960.5636.

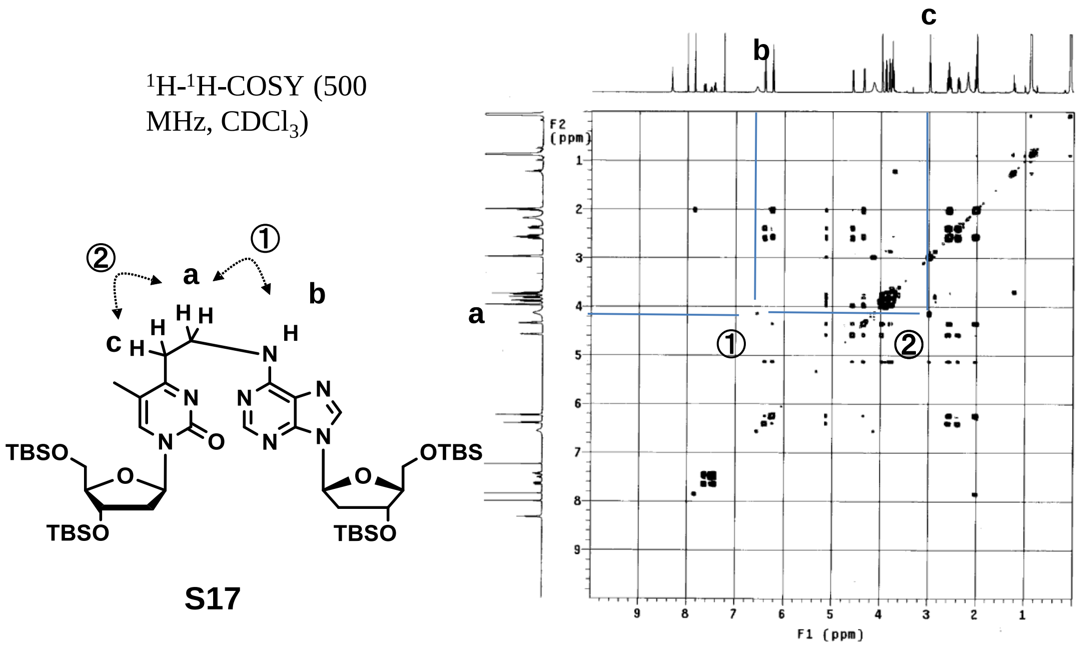

Figure S1. ^{1}H-^{1}H-COSY of the authentic adduct (S17).

A THF solution of TBAF (1.0 M solution, 69 µL, 0.07 mmol) was added to a solution of **S17** (15.8 mg, 0.02 mmol) in dry THF (0.5 mL) at room temperature under an argon atmosphere. After 30 min, the reaction mixture was concentrated under reduced pressure. The residue was purified by silica gel chromatography ($CHCl_3$-MeOH = 1:0 to 10:1, v/v) and HPLC to give the **17** as a colorless oil (1.8 mg, 3.57 µmol, 22%): ^{1}H-NMR (500 MHz, CD_3OD) d (ppm) 8.29 (1H, s), 8.22 (1H, s), 8.19 (1H, s), 6.40 (1H, dd, *J* = 8.0, 6.0 Hz), 6.17 (1H, t, *J* = 6.2 Hz), 4.56 (1H, dt, *J* = 5.7, 2.5 Hz), 4.36 (1H, dt, *J* = 6.2, 4.6 Hz), 4.05 (1H, dt, *J* = 3.0, 2.5 Hz), 4.03 (2H, broad), 3.99 (1H, dt, *J* = 3.7, 3.7 Hz), 3.86-3.81 (2H, m), 3.76-3.71 (2H, m), 3.04 (2H, t, *J* = 6.6 Hz), 2.79 (1H, ddd, *J* = 13.5, 8.0, 5.7 Hz), 2.50 (1H, ddd, *J* = 13.7, 6.2, 4.6 Hz), 2.38 (1H, ddd, *J* = 13.5, 6.0, 2.8 Hz), 2.15 (1H, ddd, *J* = 13.7, 6.2, 6.2 Hz), 2.09 (3H, s); ^{13}C-NMR (125 MHz, $CDCl_3$) d (ppm) 178.3, 157.2, 156.2, 153.4, 149.2, 142.8, 141.0, 121.3, 115.3, 89.9, 89.4, 88.7, 87.1, 73.1, 71.3, 63.7, 62.2, 42.5, 41.6, 38.8, 36.2, 14.6; IR (cm^{-1}): 3323, 2927, 1650, 1620, 1514; HR ESI MS (m/z) calcd for $C_{22}H_{29}N_7O_7$ $[M+H]^+$ 504.2201, found: 504.2244.

Scheme S3. Synthesis of the authentic tymidine adduct

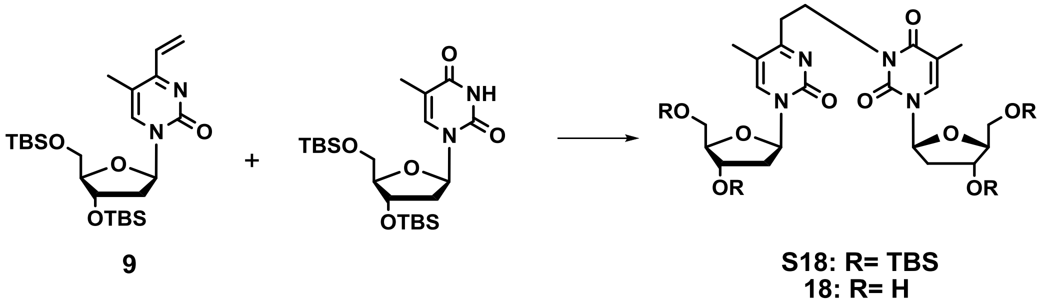

S18: R= TBS
18: R= H

$Pd(PPh_3)_4$ (19 mg, 0.02 mmol), LiBr (17 mg, 0.20 mmol) , K_2CO_3 (13 mg, 0.10 mmol) and 2,4,6-trivinylcyclotriboroxane pyridine complex (55 mg, 0.23 mmol) were added to a

solution of **7** (120 mg, 0.16 mmol) in 1,4-dioxane (1.5 mL) and H_2O (0.5 mL) at 120 ^{o}C under an argon atmosphere, and the mixture was stirred for 1 h. An aqueous HCl (2 M, 50 μL, 0.1 mmol) and 3',5'-*O*-di(TBS)-thymidine (115 mg, 0.24 mmol) were added at room temperature, and then the mixture was stirred for an additional 2 h at the same temperature. The reaction mixture was quenched with saturated aqueous $NaHCO_3$ (20 mL),extracted with $CHCl_3$ (20 mL×2), then washed with brine. The organic layer was dried over Na_2SO_4, filtered, and concentrated under reduced pressure. The residue was purified by silica gel chromatography ($CHCl_3$-MeOH = 1:0 to 100:1 to 50:1 to 20:1, v/v) to give **S18** as a yellow oil (16.2 mg, 0.02 mmol, 10% 2 steps); ^{1}H-NMR (400 MHz, $CDCl_3$)δ(ppm): 7.83 (1H, s), 7.41 (1H, s), 6.34 (1H, t, *J* = 6.1 Hz), 6.24 (1H, t, *J* = 6.1 Hz), 4.40-4.37 (2H, m), 4.35 (2H, t, *J* = 7.6 Hz), 3.96 (1H, dt, *J* = 3.7, 3.7 Hz), 3.91－3.73 (5H, m), 2.90 (2H, t, *J* = 7.6 Hz), 2.54 (1H, ddd, *J* = 11.0, 6.1, 4.3 Hz), 2.27－2.22 (1H, m), 2.15－1.99 (2H, m), 2.04 (3H, s), 1.90 (3H, s), 0.91 (9H, s), 0.90 (9H, s), 0.88 (9H, s), 0.87 (9H, s), 0.10 (3H, s), 0.09 (3H, s), 0.06 (3H, s), 0.06 (3H, s); ^{13}C-NMR (125 MHz, $CDCl_3$) d (ppm) 175.8, 163.4, 155.1, 150.7, 139.5, 133.5, 111.9, 110.0, 88.2, 87.7, 86.9, 85.4, 72.2, 71.4, 63.0, 62.5, 42.4, 41.3, 38.5, 33.4, 25.9, 25.8, 25.7, 18.4, 18.0, 14.7, 13.2, -4.5, -4.6, -4.8, -4.9, -5.4, -5.5; IR (cm^{-1}): 2928, 2857, 1706, 1668; HR ESI MS (m/z) calcd for $C_{46}H_{87}N_4O_9Si_4$ $[M+H]^+$ 951.5545, found 951.5585.

A THF solution of TBAF (1.0 M solution, 0.88 mL, 0.88 mmol) was added to a solution of **S18** (200 mg, 0.21 mmol) in dry THF (1.2 mL) at room temperature under an argon atmosphere, and the mixture was stirred for 20 min, the reaction mixture was concentrated under reduced pressure. The residue was purified by silica gel chromatography

($CHCl_3$-MeOH = 1:0 to 100:1 to 50:1 to 20:1 to 10:1 to 3:1, v/v) to give the **18** as a red oil (47.7 mg, 0.10 mmol, 46%); ^{1}H-NMR (500 MHz, CD_3OD)δ(ppm): 8.30 (1H, s), 7.82 (1H, d, *J* = 1.2 Hz), 6.22 (1H, t, *J* = 6.6 Hz), 6.16 (1H, t, *J* = 6.2 Hz), 4.38 (1H, dt, *J* = 3.7, 3.4 Hz), 4.37 (1H, dt, *J* = 4.4, 3.9 Hz), 4.31-4.28 (2H, m), 3.99 (1H, dt, *J* = 3.9, 3.7 Hz), 3.89 (1H, dt, *J* = 3.7, 3.4 Hz), 3.85 (1H, dd, *J* = 12.1, 3.0 Hz), 3.79 (1H, dd, *J* = 12.1, 3.4 Hz), 3.75 (1H, dd, *J* = 12.1, 3.7 Hz), 3.71 (1H, dd, *J* = 12.1, 3.9 Hz), 2.97−2.89 (2H, m), 2.50 (1H, ddd, *J* = 13.7, 6.2, 4.6 Hz), 2.25 (1H, ddd, *J* = 13.7, 6.2, 3.9 Hz), 2.22-2.13 (2H, m), 2.15 (3H, s), 1.88 (3H, d, *J* = 1.2 Hz); ^{13}C-NMR (125 MHz, CD_3OD) d (ppm): 177.9, 165.3, 157.0, 152.3, 142.9, 136.6, 115.2, 110.6, 89.5, 88.9, 88.7, 87.3, 72.0, 71.3, 62.7, 62.3, 42.6, 41.4, 39.6, 14.5, 13.1; IR (cm^{-1}) 3275, 2930, 2869, 1697, 1643, 1511, 1468; HR ESI MS (m/z) calcd for $C_{22}H_{31}N_4O_9$ $[M+H]^+$ 495.2086, found 495.2037.

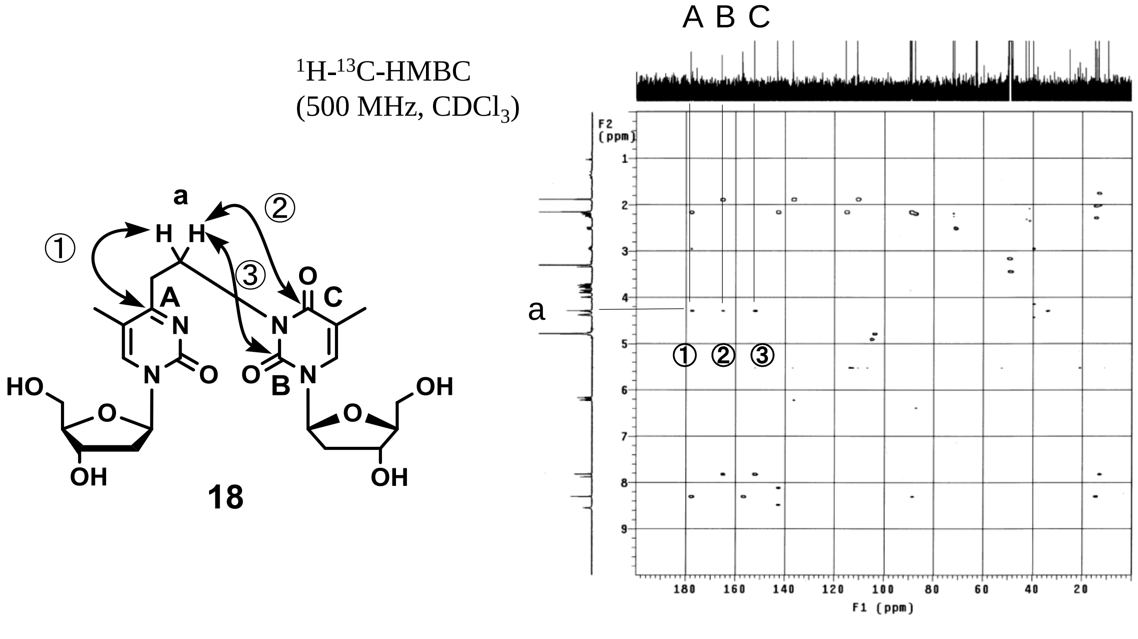

Figure S2. ^{1}H-^{13}C-HMBC of the authentic adduct (**18**).

Oligonucleotide Synthesis by a DNA/RNA Synthesizer

All oligonucleotides were synthesized at 1 μmol scale by a standard *β*-cyanoethyl phosphoramidite chemistry. The 5'-terminal dimethoxytrityl-bearing ODN was removed from the solid support by treatment with 45 mM K_2CO_3 and 10 mM $C_8H_{17}SH$ in dry MeOH (1 mL). After 1h, 0.1 M TEAA Buffer (1 mL) was added to the solution, and the mixture was purified by reverse-phase HPLC (Column: nacalai tesque: COSMOSIL 5C18-AR-II, 10×250 mm; Solvent: A: 0.1 M TEAA buffer, B: CH_3CN, B: 10% to 40%/20 min, linear gradient; Flow rate: 3.0 ml/min; monitored by UV detector at 254 nm). The dimethoxytrityl group of the purified ODN was cleaved with 5% AcOH and the mixture was additionally purified by HPLC with the same elution method. MALD-TOF-MS data are summarized in Table S2.

The generation of the vinyl group in the oligonuculeotide by MMPP oxidation and following NaOH treatment.

The procedure for the preparation of ODN3 is described as a general procedure. A solution of magnesium monoperoxyphtalate hexahydrate (MMPP, 1 mM, 0.24 μL, 240 pmol) in a carbonate buffer (1 mM, 0.24 μL, 240 pmol) adjusted to pH 10 was added to a solution of **ODN1** (190 μM, 0.42 μL, 80 pmol) at room temperature. After 30 min, NaOH (4 M, 0.2 μL) was added, and the mixture was left for an additional 1 min. The mixture was adjusted to pH 7 by the addition of AcOH (25%, 0.17 μL) to give **ODN3**. MALDI-TOF/MS (*m/z*) calcd 4736.70 ($[M\text{-}H]^{-1}$), found 4736.77. **ODN3** was used without HPLC purification. Figure S3 illustrates the HPLC change for the production of ODN**3** from ODN**1**.

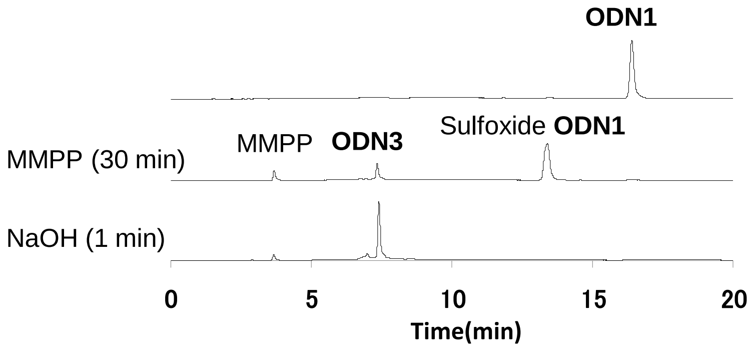

Figure S3. HPLC Analysis of the preparation of ODN1.

Reduction of ODN3 (R=CH_3) with $NaBH_4$. An aqueous solution of $NaBH_4$ (1 M, 0.3 μL) was added to a solution of **ODN3** (77.5 μM, 2.58 μL, 200 pmol) at pH 2~3 at room temperature. After 30 min, the resulting mixture was purified by reverse-phase HPLC (Column: SHISEIDO C18, 4.6×250 mm; Solvent: A: 0.1 M TEAA buffer, B: CH_3CN, B: 10% to 15%/20 min, linear gradient; Flow rate: 1.0 ml/min; monitored by UV detector at 254 nm) to give reduced **ODN3**. MALDI-TOFMS (*m/z*) **ODN3**: calcd 4738.72, found 4738.78.

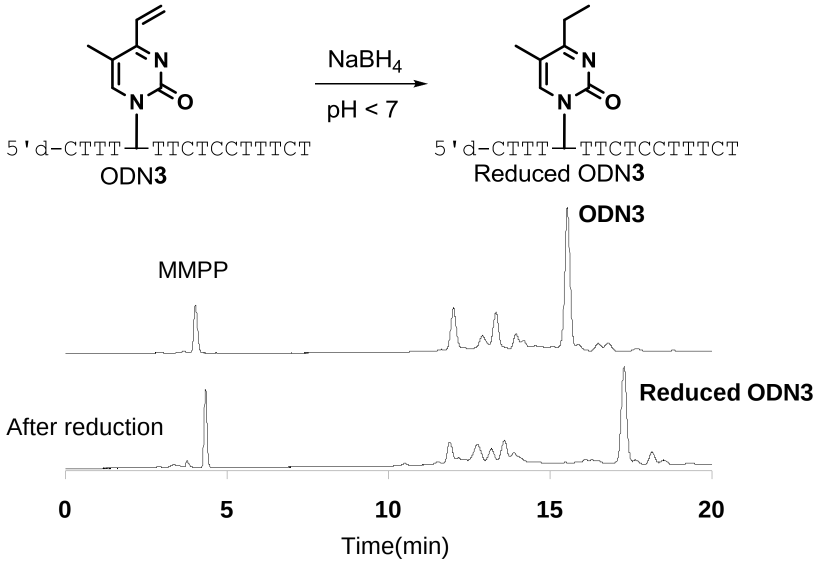

Figure S4. HPLC Analysis of the reduction of **ODN3**.

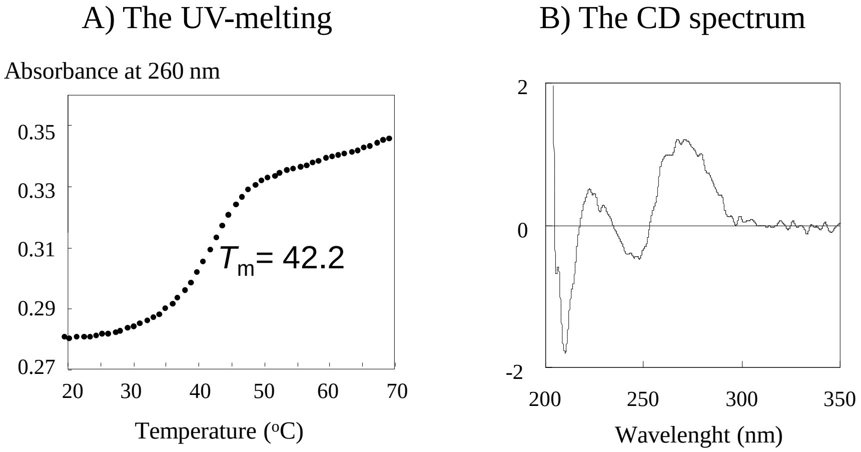

Figure S5. UV melting curve (A) and the CD spectra (B) of the duplex formed with the reduction of **ODN1**. The UV melting curve was measured using 1μM duplex in 50 mM MES buffer containing 100 mM NaCl at pH 7 and the CD spectra was measured using 2.5 μM duplex in 50 mM MES buffer containing 100 mM NaCl at pH 7 and 25 °C.

Cross-link formation with the RNA and DNA substrate. The reaction between **ODN3 (R=CH_3)** and **RNA1** is described as a general procedure. The mixture of FAM-labeled **RNA1** (1 μM) and **ODN3** (10 μM) in MES buffer (50 mM) containing 100 mM NaCl at pH 7 was incubated at 37 °C. The cross-linking reaction at pH 9 was performed using a solution in 50 mM carbonate buffer instead of MES buffer. The reaction was stopped by mixing with a loading buffer (95% formamide, 20 mM EDTA, 0.05% bromophenol blue, 0.05% xylene cyanol) at the appropriate time, and the mixture was heated at 90 °C for 3 min. The mixture was subjected to the gel electrophoresis using 15% denatured polyacrylamide gel containing urea (7 M) with TBE buffer at 250 V for 2 h. The FAM-labeled bands of the gel were visualized and quantified by a luminescent image analyzer LAS-4000 (FujiFilm) with λ_{ex}=494 nm, λ_{em}=518 nm. The yield was obtained from the fluorescent intensity of the FAM-labeled **RNA1** and the cross-linked bands. Examples

are shown in Figure 1. The reactions with the **DNA-S1**(X) substrates were performed under the same conditions except that 1M NaCl was used in the buffer at pH 7 and 9 (Figure S7).

Kinetic analysis of the cross-link formation

The cross-link reaction was performed at 5, 10, 15, 20, 25 30 and 37 ^{o}C under the same conditions as described above, and the yields were obtained using ODN**3** or **4** and RNA1 as described above. The obtained yields were analyzed based on the first-order kinetics to produce rate constants (*k*) of the cross-link formation at different temperature. Linear Arrhenius plots were obtained by plotting ln(*k*) vs 1/ T (K) (Figure S6). The obtained kinetic parameters are summarized in Table S1. Conformations calculated by MO are also shown in Figure S6.

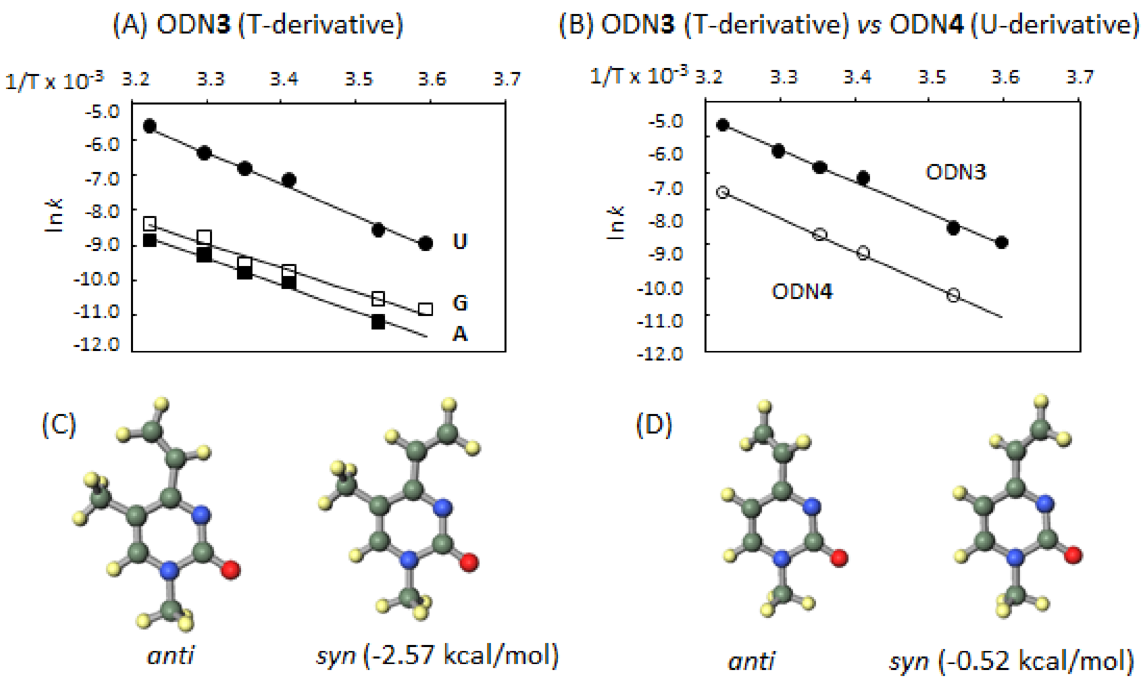

Figure S6. The Arrhenius plots of the rete constants for the cross-link formation using ODN**3** (A). Comparison between ODN**3** and ODN**4** (B). Conformations calculated by MO (B3LYP/6-31G*) for T-vinyl (C) and those for U-vinyl (D).

Table S1. Kinetic parameters for the cross-link formation with ODN**3** and ODN**4**.

Probe/Base	*Ea*(J)	*Ea*(kcal)	$\Delta G^{\ddagger}$(J)	$\Delta H^{\ddagger}$(J)	$\Delta S^{\ddagger}$(J/K)	*A*
ODN**3** (CH_3) / U	7.47E+04	17.9	7.97E+04	7.23E+04	-2.53E+01	1.33E+10
ODN**3** (CH_3)/ G	5.59E+04	13.4	9.56E+04	5.35E+04	-1.43E+02	5.57E+06
ODN**3** (CH_3)/ A	6.31E+04	15.1	9.75E+04	6.06E+04	-1.24E+02	6.52E+06
ODN**4** (H) / U	7.85E+04	18.8	9.44E+04	7.60E+04	-6.20E+01	8.65E+09

$\ln k = -Ea/RT + \ln A$, $\Delta G^{\ddagger} = -RT\ln K$, $\Delta G^{\ddagger} = \Delta H^{\ddagger} - T\Delta S^{\ddagger}$, $\Delta H^{\ddagger} = Ea - RT$

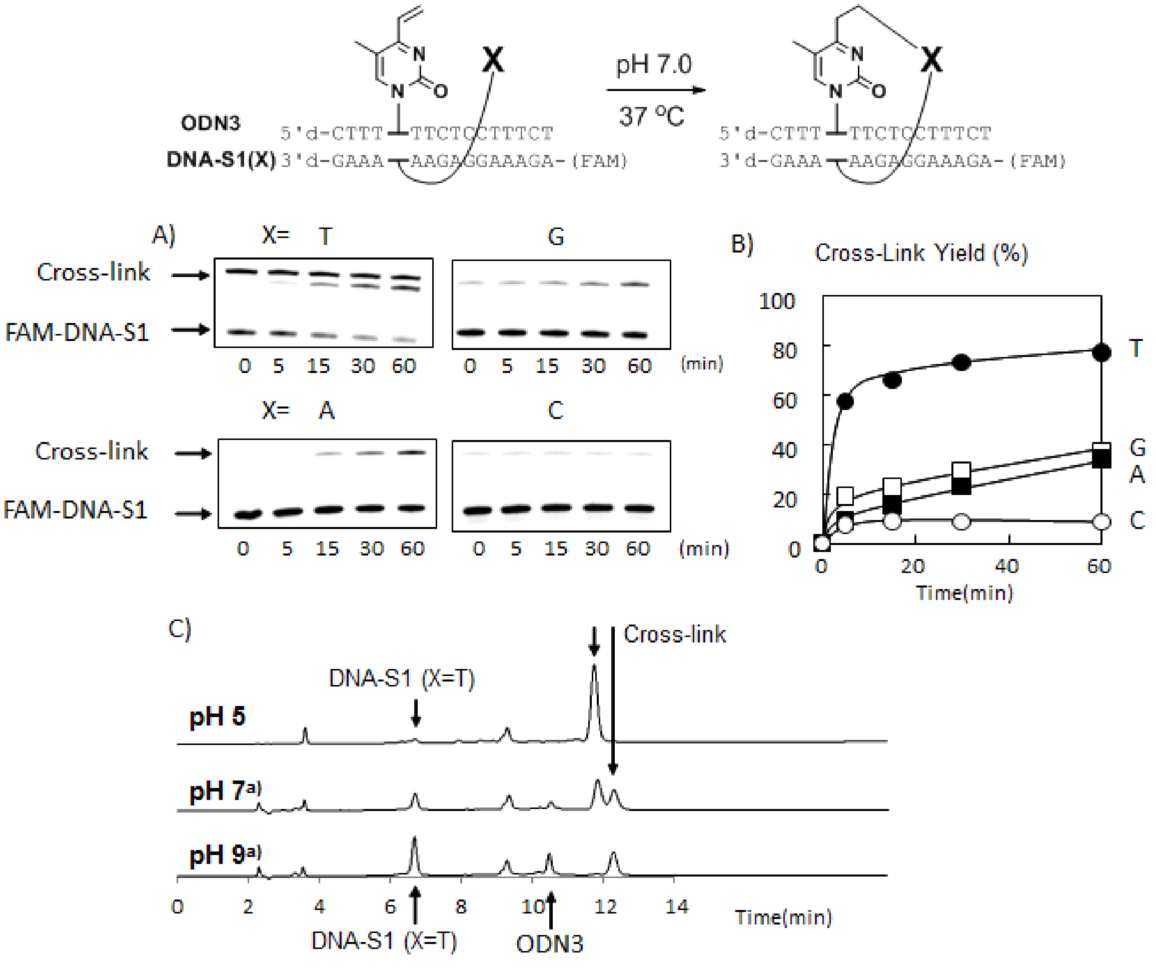

Figure S7. Cross-link formation with the **DNA-S1** substrate. (A) The gel images of the reactions. (B) Time-course of the cross-linking yields (%) obtained from the gel analysis. (C) The HPLC analysis of the cross-linking reaction using the non-FAM labeled **DNA-S1** substrate.

The HPLC analysis of the reaction mixture using non FAM-labeled RNA2 (Figure 4)

RNA2 (10 μM) and **ODN5** (15 μM) were reacted in a MES buffer (100 mM NaCl, 50 mM) at pH 7 and 37 °C. The reaction mixture was analyzed by reverse-phase HPLC (Column: SHISEIDO C18, 4.6×250 mm; solvent A 0.1 M TEAA buffer, B CH_3CN, B 10% to 20%/20 min, linear gradient; flow rate 1.0 ml/min; monitored by UV detector at 254 nm. Results are summarized in Figure 4.

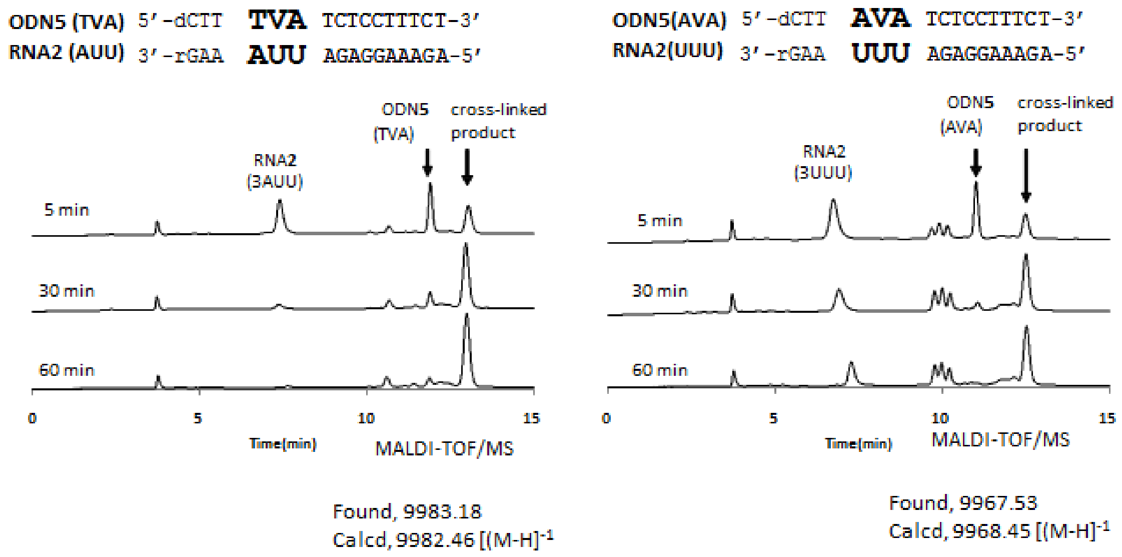

Figure S8. The HPLC charts of the cross-linking reaction. The cross-linked products were isolated and subjected to the enzymatic digestion to analyze the ratio of the A- and U-crosslink. The obtained ratios are summarized in Figure 4.

The enzymatic digestion of the isolated cross-linked products and their HPLC analysis.

The cross-linked products were isolated by HPLC under the conditions as described above, and the eluents were freeze-dried. The residue was mixed with BAP(0.5 u/μL, 1 μL,

0.5 u), VPDE (0.1 u/μL, 1 μL, 0.1 u) and P1 nuclease (0.4 u/μL, 4 μL, 1.6 u) in alkaline phosphatase buffer (500 mM Tris-HCl, 10 mM $MgCl_2$, 1 μL, pH 9, 0.5 μmol Tris-HCl, 0.01 μmol $MgCl_2$) and super purified water (7 μL) , and the mixture was incubated at 37 ^{o}C for 2 -24 h. The reaction mixture was analyzed by reverse-phase HPLC (Column: SHISEIDO C18, 4.6×250 mm; Solvent: A: 50 mM $HCOONH_4$ buffer, B: CH_3CN, B: 10% to 15%/20 min, linear gradient; Flow rate: 1.0 ml/min; monitored by UV detector at 254 nm). The results are shown in Figures 3.

Molecular Modeling of the ODN3/RNA1 duplex containing 2.

The structure was built by modifying pdb479, and only the local conformation of the flanking base pair was optimized by AMBER force field on HyperChem 7.5. The neighboring base pairs are shown in space-filling model and other parts are shown in tubes (Figure S9). In the duplex having a uridine at the opposite site to **2**, the vinyl group of **2** is in van der Waals contact with the 6-amino group of the adenine base at the 5' side.

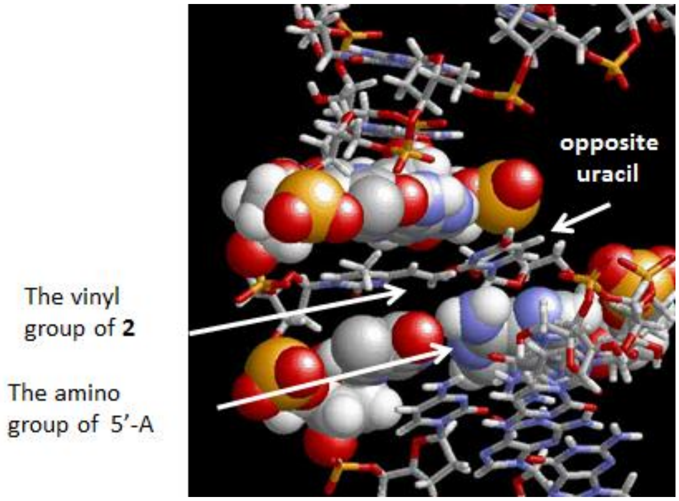

Figure S9. Molecular modeling of the **ODN3**/**RNA1** duplex having a uridine at the opposite site to **2**.

Cross-link formation in the triplex DNA.

The data shown in Figure 8 was obtained by incubating a solution of the duplex DNA (5 μM each DNA**1** and DNA**2**) and ODN**3** (25 μM) in MES buffer (100 mM NaCl, 50 mM MES) containing $MgCl_2$ (10 mM) at pH 5 and 25 ^{o}C. The reaction mixture was stopped by mixing with a loading buffer (95% formamide, 20 mM EDTA, 0.05% bromophenol blue, 0.05% xylene cyanol) at the appropriate time, and the mixture was heated at 90 ^{o}C for 3 min. The above mixtures were subjected to the gel electrophoresis using 15% denatured polyacrylamide gel containing urea (7 M) with TBE buffer at 250 V for 2 h. and the intensity of FAM-labeled bands were quantified by a luminescent image analyzer LAS-4000 (Fujifilm). The reaction was performed at different pH using OND**3** or ODN**6.**

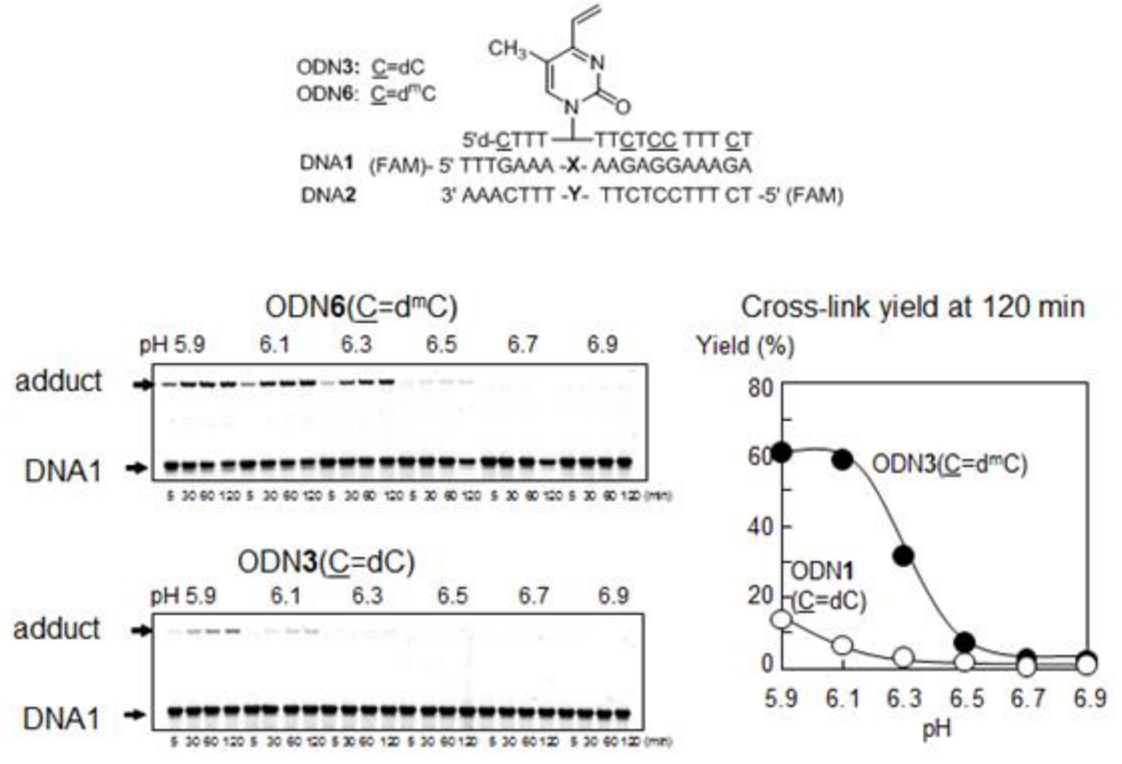

Figure S10. The cross-link formation at different pH using **ODN3** and **ODN6.**

Table S2. MALDI-TOFMS Data（m/e [M-H]$^-$）

ODN or DNA	Figures	Notes	calcd	found
ODN**1**	Scheme 1	5’-DMTr, *S*-octyl	5184.94	5184.91
ODN**1**	Fig. S3	*S*-octyl	4882.81	4882.81
ODN**1**	Fig. S3	*S*-octyloxide	4898.80	4898.91
ODN**2**	Scheme 1	*S*-octyl	4868.80	4868.71
ODN**3**	Scheme 1	T-vinyl derivative (**2**)	4736.70	4736.77
ODN**4**	Scheme 1	U-vinyl derivative (**3**)	4722.69	4722.52
CL with ODN**3**	Fig. 1&2	Target RNA**1**, X=A	10019.50	10019.50
CL with ODN**3**	Fig. 1&2	Target RNA**1**, X=G	10035.49	10035.42
CL with ODN**3**	Fig. 1&2	Target RNA**1**, X=C	9995.48	9998.21
CL with ODN**3**	Fig. 1&2	Target RNA**1**, X=U	9996.46	9996.08
CL with ODN**4**	Fig. 1&2	Target RNA**1**, X=U	9982.93	9982.50
Reduced ODN**3**	Fig. S4	Ethyl	4738.72	4738.78
CL with ODN**3**	Fig. S7	Target DNA**1**, X=A	9763.66	9763.90
CL with ODN**3**	Fig. S7	Target DNA**1**, X=G	9779.65	9779.16
CL with ODN**3**	Fig. S7	Target DNA**1**, X=C	9739.64	9737.62
CL with ODN**3**	Fig. S7	Target DNA**1**, X=T	9754.64	9754.67
ODN**5**	Fig. 4	5’ A-**2**-T	4745.72	4737.45
ODN**5**	Fig. 4	5’ G-**2**-T	4761.71	4761.93
ODN**5**	Fig. 4	5’ C-**2**-T	4721.70	4721.79
ODN**5**	Fig. 4, Fig. S8	5’ T-**2**-A	4745.72	4746.20
ODN**5**	Fig. 4, Fig. S8	5’ A-**2**-A	4754.74	4754.79
ODN**5**	Fig. 4	5’ G-**2**-A	4770.73	4770.95
ODN**5**	Fig. 4	5’ C-**2**-A	4730.72	4731.04
ODN**5**	Fig. 4	5’ T-**2**-G	4761.71	4761.85
ODN**5**	Fig. 4	5’ A-**2**-G	4770.73	4770.83
ODN**5**	Fig. 4	5’ G-**2**-G	4787.72	4787.10
ODN**5**	Fig. 4	5’ C-**2**-G	4746.71	4747.31
ODN**5**	Fig. 4	5’ T-**2**-C	4721.70	4722.09
ODN**5**	Fig. 4	5’ A-**2**-C	4730.72	4730.90
ODN**5**	Fig. 4	5’ G-**2**-C	4746.71	4747.14
ODN**5**	Fig. 4	5’ C-**2**-C	4706.70	4707.27
CL with ODN**5**	Fig. 4	Target RNA**2**, 3’ UUA	9982.46	9982.59
CL with ODN**5**	Fig. 4	Target RNA**2**, 3’ CUA	9997.47	9996.47
CL with ODN**5**	Fig. 4	Target RNA**2**, 3’ GUA	9997.46	9997.02
CL with ODN**5**	Fig. 4, Fig. S8	Target RNA**2**, 3’ AUU	9982.46	9983.18
CL with ODN**5**	Fig. 4, Fig. S8	Target RNA**2**, 3’ UUU	9968.45	9967.53
CL with ODN**5**	Fig. 4	Target RNA**2**, 3’ CUU	9983.46	9982.20
CL with ODN**5**	Fig. 4	Target RNA**2**, 3’ GUU	9983.45	9983.64
CL with ODN**5**	Fig. 4	Target RNA**2**, 3’ AUC	9997.47	9997.15

Table S2. MALDI-TOFMS Data (continued)

ODN or DNA	Figures	Notes	calcd	found
CL with ODN**5**	Fig. 4	Target RNA**2**, 3’ UUC	9983.46	9983.01
CL with ODN**5**	Fig. 4	Target RNA**2**, 3’ CUC	9998.47	9998.24
CL with ODN**5**	Fig. 4	Target RNA**2**, 3’ GUC	9998.46	9998.48
CL with ODN**5**	Fig. 4	Target RNA**2**, 3’ AUG	9997.47	9998.41
CL with ODN**5**	Fig. 4	Target RNA**2**, 3’ UUG	9983.45	9984.29
CL with ODN**5**	Fig. 4	Target RNA**2**, 3’ CUG	9998.46	9998.79
CL with ODN**5**	Fig. 4	Target RNA**2**, 3’ GUG	9998.45	9998.97
ODN**6**	Fig. 5	5’ DMTr, *S*-octyl	5255.04	5255.30
ODN**6**	Fig. 5	*S*-octyl	4952.91	4952.91
ODN**6**	Fig. 5	T-vinyl derivative (**2**)	4806.81	4807.62
CL with ODN**3**	Fig. 6	Target DNA**1**, X=A	11542.90	11542.55
